# Structural Improvement of the Allosteric ALOX15 Inhibitor Octyl (*N*-(5-(1*H*-Indol-2-yl)-2-methoxyphenyl)sulfamoyl)carbamate

**DOI:** 10.3390/molecules31142544

**Published:** 2026-07-22

**Authors:** Viktor Gavrilyuk, Vladislav Aksenov, Kirill Petrov, Angelina V. Kurchatova, Dmitriy Bortnevskij, Alexander Zhuravlev, Alexey Golovanov, Hartmut Kuhn, Igor Ivanov

**Affiliations:** 1 Lomonosov Institute of Fine Chemical Technologies, MIREA—Russian Technological University, Vernadskogo pr. 86, 119571 Moscow, Russia; viktor_gavril@inbox.ru (V.G.); aksenov_v@mirea.ru (V.A.); kirillpetrovv.2015@yandex.ru (K.P.); akurchatova07@mail.ru (A.V.K.); dbortnevskij@yandex.ru (D.B.); alekszhur95@yandex.ru (A.Z.); golovanov_a@mirea.ru (A.G.); 2 Institute of Biochemistry, Charite—University Medicine Berlin, Corporate Member of Free University Berlin, Humboldt University Berlin and Berlin Institute of Health, Charitéplatz 1, 10117 Berlin, Germany; hartmut.kuehn@charite.de

**Keywords:** heterocyclic compounds, lipoxygenases, inhibitors, allostery, lipid metabolism

## Abstract

Octyl (*N*-(5-(1*H*-indol-2-yl)-2-methoxyphenyl)sulfamoyl)carbamate has previously been characterized as substrate-specific inhibitor of the linoleic acid oxygenase activity of mammalian ALOX15 orthologs. Here we aimed at optimizing the inhibitory properties of this compound by three different chemical modifications: (i) replacement of the indole core by a phenylpyrrole; (ii) introduction of hydrophilic residues into the aliphatic hydrocarbon chain of the lead compound or by replacing this building block by a triethylene glycol moiety; (iii) replacement of the sulfamoylcarbamate group by a sulfonamide. The inhibitory potencies of the modified compounds for pure rabbit ALOX15 were quantified by in vitro inhibitory assays, and our data indicate that the replacement of the rigid indole core induced a partial loss in the inhibitor’s potency. The introduction of a hydrophilic group into the aliphatic hydrocarbon chain or its replacement by a triethylene glycol moiety improved the solubility of the compound in aqueous solution, but reduced the inhibitor potency by more than one order of magnitude. Finally, the replacement of the sulfamoylcarbamate moiety by sulfonamide improved the substrate selectivity of the inhibitor for rabbit and human ALOX15. The new compounds were highly potent for human and rabbit ALOX15, but did not inhibit human ALOX15B and were less effective for mouse Alox15 (ortholog specificity).

## 1. Introduction

**Figure 1:**
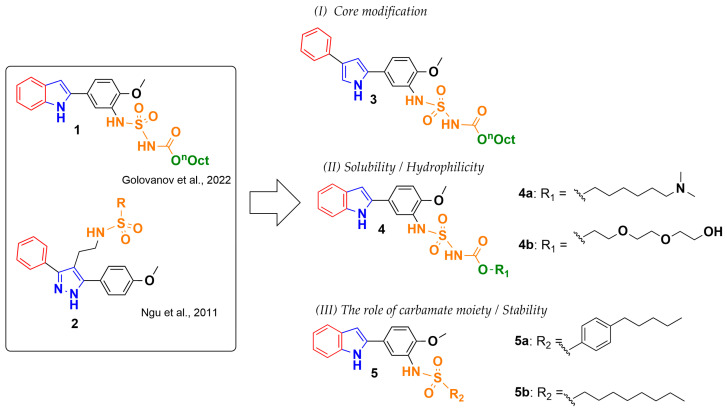
Structure of selective inhibitors of the linoleate–oxygenase activity and targeted modifications of compound **1**: (I) core modification; (II) solubility and hydrophilicity; (III) the role of carbamate moiety and stability [16,17].

Mammalian 15-Lipoxygenases (ALOX15) are lipid-peroxidizing enzymes that have been implicated in cell differentiation [1,2,3] and ferroptosis [4,5], but also in the pathogenesis of atherosclerosis [6,7] and other inflammatory disorders [8,9,10]. These enzymes oxygenate polyunsaturated fatty acids (PUFAs) to their corresponding hydroperoxy derivatives [11]. The dual functionality of these enzymes in different types of cancer and their involvement in the pathogenesis of inflammation made these enzymes promising targets for pharmacological research. Mammalian ALOX15 are allosteric enzymes [12]. Although the molecular basis for the allosteric character of ALOX15 remains a matter of discussion, an inter-monomer communication within the ALOX15 dimer has previously been suggested [13]. According to this scenario, the binding of an inhibitor at the active site of one monomer within the ALOX15 dimer induces conformational alterations in the other monomer [14] and, thus, may modify the rate constants of the rate-limiting step of PUFA oxygenation. However, this inter-monomer communication is not a one-way street. In fact, the binding of a substrate fatty acid at the active site of one ALOX15 monomer may also modify the structure of the inhibitor–enzyme complex, forcing the inhibitor to adopt a different binding mode [15]. In other words, the inhibitory potency of allosteric ALOX15 inhibitors depends on the chemical structure of the PUFA substrate. We recently reported that octyl (*N*-(5-(1*H*-indol-2-yl)-2-methoxyphenyl)sulfamoyl)carbamate (**1**) is a substrate-selective inhibitor that modifies the linoleate (LA)-oxygenase activity of human and rabbit ALOX15 with an IC_50_ in the lower nanomolar range [16]. At these concentrations, the oxygenation of arachidonic acid (AA) was not affected. Although the molecular basis for the substrate specificity of the inhibitory activity has not been fully clarified, we found that the presence of the rigid 4-MeO-aryl moiety in the core pharmacophore of compound **1** might be important for inhibitor binding at the active site of the allosteric monomer. Moreover, the NH-group of the indole moiety was suggested to form a hydrogen bond with the Fe(III)-OH¯-cofactor, and NH-to-O exchange in the main core pharmacophore reduced the degree of substrate selectivity [15]. 5-(4-Methoxyphenyl)-3-phenyl-1*H*-pyrazol-based sulfonamide (compound **2**) (Figure 1) [17] did also function as a substrate-selective ALOX15 inhibitor, and this finding suggests the possibility of the replacement of condensed indole rings with a separated ring assembly.

The present study was aimed at further optimizing the inhibitory potency of compound **1**. For this purpose, we modified the structure of **1** in three different ways (Figure 1), as follows: (i) We replaced the indole core of **1** with a 4-phenyl-1*H*-pyrrol-2-yl fragment. For this modification, we synthesized a new compound, **3,** that has not been reported before. (ii) Most ALOX inhibitors are hydrophobic compounds that exhibit a low degree of water solubility. The introduction of a hydrophilic tail into the core pharmacophore of **1** (compounds **4a**–**b**) is likely to decrease its lipophilicity and improve its water solubility. However, this structural modification might also impact the inhibitory potency. (iii) The sulfamoylcarbamate moiety of compound **1** may undergo degradation in aqueous solutions in the presence of Fe^3+^, and this reaction might induce a reduction of Fe^3+^-ALOX15 to its ferrous form [16]. Since the sulfamoylcarbamate moiety is a hybrid structure, which often serves as a sulfonamide “prodrug” [18], we replaced it with a sulfonamide residue to obtain compounds **5a**–**b**. Finally, the inhibitory potencies of the newly synthesized compounds **3**–**5** were tested against pure recombinant rabbit ALOX15, with LA and AA as substrates, and the mechanism of inhibition was investigated. The selected inhibitors were also tested for their inhibitory potencies for human ALOX15, its mouse ortholog (mouse Alox15) and against human ALOX15B.

## 2. Results and Discussion

### 2.1. Description of the Synthesis Procedure

#### 2.1.1. Modification of the Indole Scaffold

**Scheme 1:**
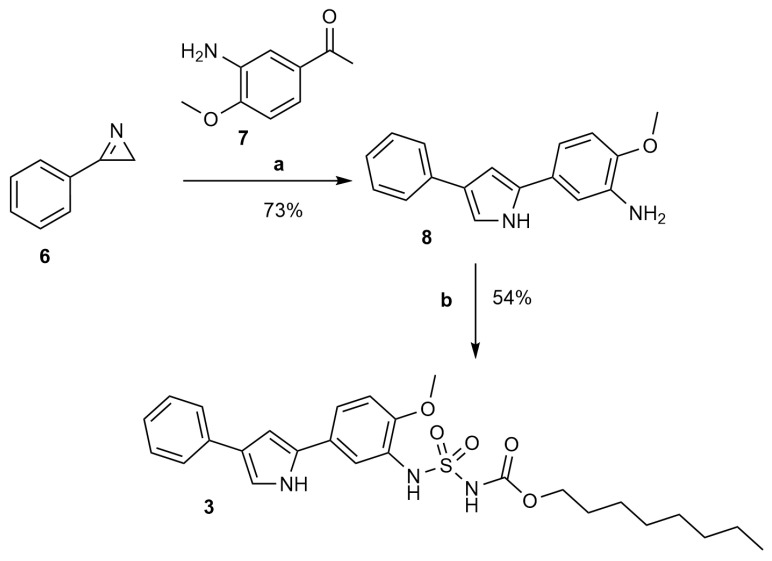
Synthesis of compound **3**. Reaction conditions: (a) NaH, DMSO, rt, overnight; (b) CSI, TEA, octan-1-ol (**9**), DCM, rt, 3 h.

Among the methods available for the preparation of 2,4-diphenyl-1*H*-pyrroles [19,20], the synthetic pathway via 3-phenyl-2*H*-azirine appears to be the most suitable. 3-Phenyl-2*H*-azirine was synthesized in a four-step procedure from styrene, as described elsewhere [19,21]. The key synthetic step for the preparation of compound **3** involves the reaction of phenylazirine (**6**) with substituted acetophenone **7** in the presence of sodium hydride (Scheme 1), yielding 2,4-diphenyl-1*H*-pyrrole **8** (73%). The target compound **3** was obtained by the reaction of the amine **8** with chlorosulfonyl isocyanate (CSI) and octan-1-ol (**9**) in the presence of triethylamine [16], with a 54% yield after purification by preparative RP-HPLC. The degree of purity of compound **3** was 99.0%.

#### 2.1.2. Modification of Hydrophilic Tail

Different analogues of compound **1** with confirmed inhibitory activity against mammalian ALOX15 orthologs [15,16] are highly hydrophobic, and thus, their water solubility is somewhat limited. To improve the water solubility of ALOX inhibitors [22], in vitro ALOX activity assays have sometimes been carried out in the presence of detergents (0.02% Tween 20) [23,24]. Unfortunately, detergents do not only enhance the solubility of the inhibitors, but they also improve the availability of the substrate fatty acids. Moreover, detergents may also impact the 3D structure of the enzymes and ALOX15 dimerization. Thus, we refrained from using detergents in our activity assays. The problem of limited water solubility can be solved at least in part by introducing polar groups into the inhibitor molecule. Another possible mode of modification would be the replacement of the hydrophobic octyl tail of compound **1** by a triethylene glycol residue.

**Scheme 2:**
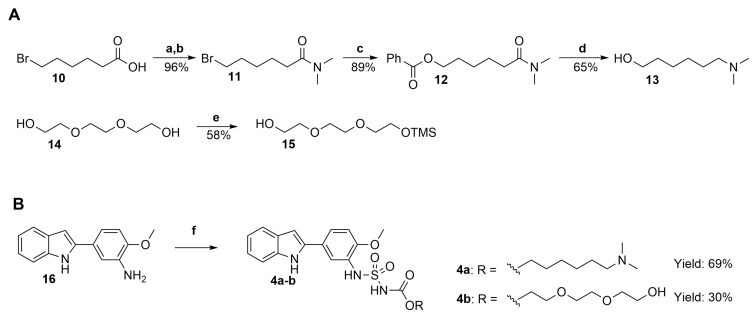
Synthesis of compounds **4a** and **4b**. (**A**) Synthesis of compounds **13** and **15**. (**B**) Synthesis of compounds **4a** and **4b** from compound **16**. Reaction conditions: (a) (COCl)_2_, DMF, DCM, rt, 2 h; (b) Me_2_NH∙HCl, DIPEA, DCM, rt, 2 h; (c) PhCOOH, K_2_CO_3_, KI, THF, 90 °C, 24 h; (d) LiAlH_4_, THF, rt, 3 h; (e) HMDS, BSTFA, THF, reflux, 16 h; (f) **12** or **14**, CSI, TEA, DCM, rt, 3 h.

Unfortunately, our initial attempt to prepare 6-(dimethylamino)hexan-1-ol (**13**) from commercially available 6-bromohexanol failed. An alternative method for the preparation of compound **13** utilizes commercially available 6-bromohexanoic acid (**10**), which may be converted to the corresponding acyl chloride followed by a reaction with dimethylamine hydrochloride in the presence of a base to give the intermediate **11** (Scheme 2A). To avoid the formation of unwanted side products during bromine-to-OH substitution, the intermediate **11** was converted to the benzoate **12** using benzoic acid in the presence of K_2_CO_3_ and KI. The further modification of this intermediate allowed the simultaneous reduction of the *N,N*-dimethylamide moiety and ester hydrolysis during the reaction with LiAlH_4_ to form the corresponding amino alcohol **13**. Thus, 6-(dimethylamino)hexan-1-ol (**13**) was obtained in a three-step procedure with an overall yield of 56%. The triethylene glycol (**14**) was used to prepare compound **15**. For this purpose, one of the OH-groups of the bifunctional alcohol **14** was protected by a TMS group using HMDS in the presence of catalytic amounts of BSTFA. The mono-TMS product **15** was prepared by silica gel chromatography, with a 58% yield.

The usual synthetic procedure for the preparation of the sulfamoylcarbamate **1** [16] involves the sequential addition of CSI, TEA and the heterocyclic amine to the corresponding alcohol. This method turned out to be unsuitable for the preparation of compound **4b** (Scheme 2B), since the hydrochloric acid, which is formed during the reaction, hydrolyzes the mono-TMS product **15**. Therefore, we modified the synthetic protocol as described below. First, a molar excess of TEA was added to CSI, followed by the addition of the alcohol and the heteroaryl substituted aniline **16** to the neutralized reaction mixture (Scheme 2B). Compound **4b** was obtained after treating the reaction mixture with water to remove the TMS-group. Compound **4a** was prepared by the standard protocol [16] and was purified by preparative RP-HPLC. The overall yields of the synthesis of compounds **4a** and **4b** were 69% and 30%, respectively, and their degrees of purity were 98.5% (**4a**) and 98.7% (**4b**).

#### 2.1.3. Replacement of the Carbamate Moiety

**Scheme 3:**
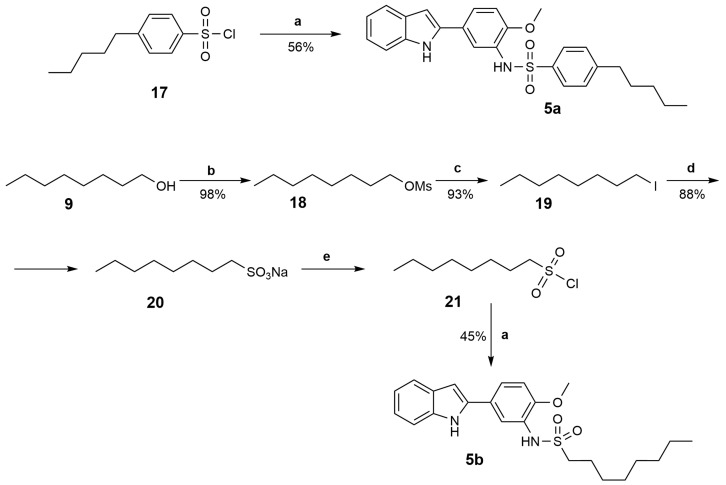
Synthesis of compounds **5a** and **5b**. Reaction conditions: (a) 5-(1*H*-indol-2-yl)-2-methoxyaniline (**16**), TEA, DCM, rt, 5 h; (b) MsCl, TEA, DCM, rt, 4.5 h; (c) NaI∙2H_2_O, Me_2_CO, reflux, 16 h; (d) Na_2_SO_3_/H_2_O, MeOH, reflux, 16 h; (e) (COCl)_2_, DMF, DCM, rt, 3 h.

Previous in silico inhibitor docking studies suggested that the C=O group of the sulfamoylcarbamate moiety might form a hydrogen bond with the backbone NH-group of Leu408 [25]. To evaluate the relevance of the carbamate moiety of compound **1** for ALOX15 inhibition, we replaced the sulfamoylcarbamate moiety of compound **1** by either a 4-amylphenylsulfonamide, which carries a planar conjugated electron-rich system, or by 1-octylsulfonamide fragments (Scheme 3). Compound **5a** was obtained by the acylation of the heteroaryl-substituted aniline **16** with sulfonyl chloride **17** in the presence of base [16]. The yield of compound **5a** after purification was 56%. Compound **5b** was prepared under similar conditions, and the corresponding sulfonyl chloride was obtained from octan-1-ol (**9**) in a four-step procedure (Scheme 3). First, octan-1-ol (**9**) was converted to the corresponding mesylate **18**, which was sequentially transformed to the corresponding iodide **19** and then to the sodium octane-1-sulfonate (**20**). The later compound was activated by the addition of oxalyl chloride to form intermediate **21,** which was applied for the reaction with the indole **16** without any purification. The yield of compound **5b** was 45%. Compounds **5a** and **5b** were purified by silica gel chromatography, and their final degrees of purity were 98.1% and 98.6%, respectively.

### 2.2. Evaluation of the Novel ALOX15 Inhibitors

#### 2.2.1. Evaluation of the Inhibitors for Rabbit ALOX15

**Figure 2:**
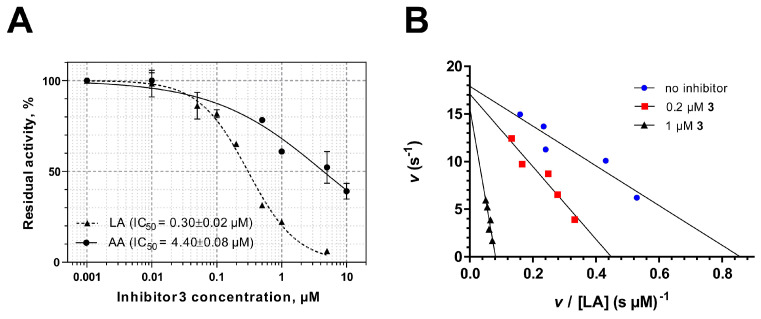
Inhibitory potency of compound **3** against rabbit ALOX15. (**A**) Dose–response curves for inhibition of the LA- and AA-oxygenase activities of pure recombinant rabbit ALOX15 by compound **3** (LA and AA concentrations 25 µM). Each data point represents the mean ± SEM of three independent activity measurements. (**B**) Eadie–Hofstee plot demonstrating the effects of compound **3** on the LA-oxygenase activity of rabbit ALOX15. Each data point represents the mean of three independent activity measurements.

The effectiveness of ALOX15 inhibition by the newly synthesized compound **3** was quantified employing the standard spectrophotometric in vitro activity assay using rabbit ALOX15 [26]. For LA and AA, the IC_50_ values of the reference compound **1** were 0.04 µM and 2.06 μM, respectively [16]. The previously determined IC_50_(LA)/IC_50_(AA) ratio was 0.019 [16], and here we confirmed this value. The replacement of the indole core with the phenylpyrrole moiety resulted in a decrease in the substrate selectivity with an IC_50_(LA)/IC_50_(AA) ratio of 0.068. The IC_50_ value of compound **3** for LA oxygenation (Figure 2A) was almost one order of magnitude higher than the corresponding value obtained for the reference compound **1.** In contrast, the IC_50_(AA) values of both inhibitors **1** and **3** were similar.

To explore the reason for the marked loss in the inhibitory potency of compound **3** for the LA-oxygenase activity of ALOX15, we carried out kinetic measurements using pure recombinant rabbit ALOX15. The mechanism of inhibition of the LA oxygenase activity of rabbit ALOX15 by compound **1** was noncompetitive, with a markedly decreased *k_ca__t_* but an unaffected K_M_ [16]. In contrast, for AA oxygenation, compound **1** induced a marked decrease in both *k_cat_* and K_M_, indicating an uncompetitive mode of inhibition [16]. The best fit for LA oxygenation suggested a mixed mode of inhibition with a K_i_ of 0.19 ± 0.07 µM (α = 5.3± 1.3, R^2^ = 0.96) (Figure 2B, Supplementary Figure S1A). In this case, a marked increase in K_M_ was observed, but *k_cat_* remained largely unaffected. For LA oxygenation, a dissociation constant ratio K_d_^EI^/K_d_^ESI^ of 0.015 was calculated suggesting a higher binding affinity of compound **3** to the unliganded enzyme (E) rather than to the enzyme–substrate complex (ES). In contrast, for AA oxygenation, a marked increase in K_M_ and a decrease in *k_cat_* was observed, and the best fit of these experimental data was obtained assuming a mixed mode of inhibition with a K_i_ of 1.91 ± 0.90 µM (α = 6.8 ± 1.3, R^2^ = 0.95) (Supplementary Figure S1B). Thus, the modes of inhibition induced by compounds **1** and **3** are mechanistically different.

Since the newly synthesized inhibitor **3** has a lower inhibitory potency than the original lead compound **1,** the rigid indole moiety of the core pharmacophore appears to be important for inhibitor binding. To explain the experimental observations, we performed in silico docking calculations of compound **3** inside the substrate-binding cavity of monomer A of the dimeric rabbit ALOX15 crystal structure (PDB entry 2P0M), as described previously [16]. Although in the highest-scoring complex, compound **3** and the reference compound **1** were located deeply inside the substrate binding cavity, the OCH_3_-group appears to be somewhat closer to the side chain of Gln596 (monomer A). A rotation of the molecule **3** relative to the central phenyl ring displaces its SO_2_-group from the side chain of Gln596 (monomer A) (Supplementary Figure S2). Gln596 of the α18 helix has been previously shown to be important for the allosteric properties of ALOX15 [27]. However, for more detailed structural information on the inhibitor–ALOX15 complex, MD simulations are required, but such studies exceed the frame of the present paper.

**Figure 3:**
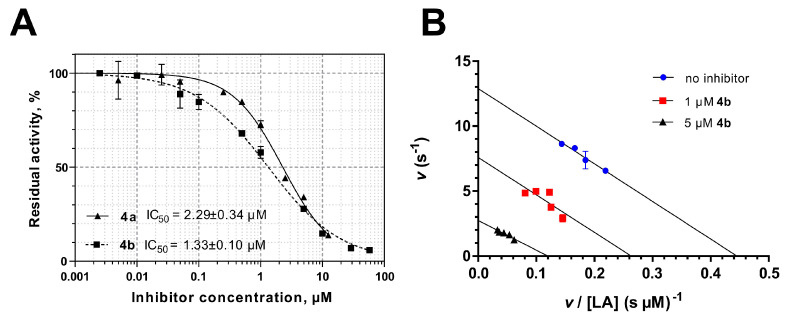
Inhibitory potency of compounds **4a** and **4b** for rabbit ALOX15. (**A**) Dose–response curves for the inhibition of the LA-oxygenase activities of the pure recombinant enzymes (25 µM LA). (**B**) Eadie–Hofstee plot demonstrating the effects of **4b** on the LA-oxygenase activity of rabbit ALOX15. Incubations were repeated at different inhibitor concentrations Each data point represents the mean ± SEM of three independent activity measurements.

Next, we determined the IC_50_ values of compounds **4a** and **4b** for the LA oxygenase activity of rabbit ALOX15 (Figure 3A), and obtained the following values: 2.29 ± 0.34 μM (**4a**) and 1.33 ± 0.10 μM (**4b**). Since the AA oxygenase activity of the enzyme was hardly impacted by the two compounds at inhibitor concentrations below 50 µM, the inhibitory activity was substrate-specific. Similar to compound **1,** the inhibition of the LA oxygenase activity by compound **4b** (Figure 3B) could best be described by a noncompetitive mode of inhibition with a K_i_ of 1.46 ± 0.08 μM (R^2^ = 0.99). This compound induced a marked decrease in *k_cat_,* but K_M_ remained largely unaffected (Supplementary Figure S3). The substrate selectivity ratio [IC_50_(LA)/IC_50_(AA)] for compound **4a** was <0.046, and for compound **4b it** was <0.027.

The introduction of a polar group increased the water solubility of compounds **4a** and **4b**. The experimental Log*p* values obtained for compounds **4a** and **4b** were 0.16 ± 0.03 and 0.49 ± 0.03, respectively (Supplementary Figure S4A). Interestingly, linear calibration curves for PBS solutions were obtained for compounds **4a** and **4b** over a concentration range of 1 µM to 100 µM. In contrast, the upper limit of linearity for compound **1** was reached at 50 µM (Supplementary Figure S4B). Thus, compounds **4a** and **4b** are more soluble in PBS than compound **1**.

Taken together, one can conclude that the replacement of the lipophilic residue in compound **1** with a more hydrophilic moiety improved the water solubility of the two inhibitors **4a** and **4b** and reduced their substrate selectivity, but strongly (more than 10-fold) impaired the inhibitor potency.

**Figure 4:**
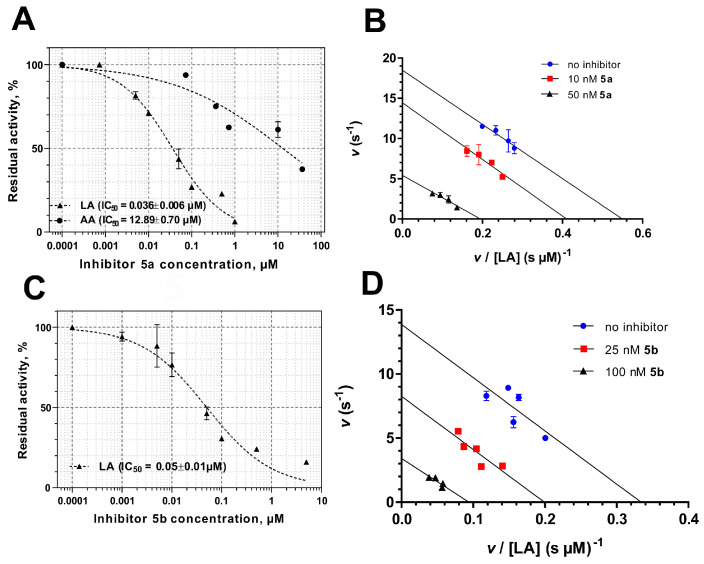
Inhibitory potency of compounds **5a** and **5b** for rabbit ALOX15. (**A**) Dose–response curves for the inhibition of the LA- and AA-oxygenase activities of pure recombinant rabbit ALOX15 by compounds **5a**. (**C**) Dose–response curve for the inhibition of the LA-oxygenase activity of recombinant rabbit ALOX15 by compound **5b**. (**B**,**D**) Eadie–Hofstee plots demonstrating the effects of **5a** and **5b** on rabbit ALOX15 when incubated with different concentrations of linoleic acid. Each data point represents the mean ± SEM of three independent activity measurements.

Next, we tested the inhibitor potencies of the sulfonamides **5a** and **5b**. The IC_50_ values for the LA oxygenase activity of rabbit ALOX15 were 0.036 ± 0.005 μM and 0.05 ± 0.01 μM for compounds **5a** and **5b**, respectively (Figure 4A,C). In contrast, for AA oxygenation, the IC_50_ values were significantly higher (12.89 ± 0.70 μM for compound **5a** and >50 μM for compound **5b**), and these data indicate the high degree of substrate selectivity of the inhibitory effect. Using LA as a substrate, both compounds **5a** and **5b** induced a marked decrease in *k_ca_*_t_, whereas the K_M_ values remained largely unaffected (Supplementary Figures S5). The inhibitor data (Figure 3B,D) were fitted to different inhibition models, and the best fits were obtained for a model of noncompetitive inhibition of LA oxygenation with a K_i_ of 27.04 ± 2.64 nM (R^2^ = 0.97) for **5a** and a Ki of 36.79 ± 2.45 nM (R^2^ = 0.98) for **5b**.

When we compared the IC_50_(LA)/IC_50_(AA) ratios for compounds **1**–**5** as suitable measures for the degree of substrate selectivity of the newly synthesized ALOX15 inhibitors (Table 1), we found that the sulfonamide **5b** exhibited the highest degree of substrate selectivity. Using the ferrozine-based system, we have previously demonstrated that the sulfamoylcarbamate **1** may degrade in aqueous solution when Fe^3+^ is present [16]. This degradation reduces Fe^3+^ ions to the ferrous (Fe^2+^) form. Fe^2+^ ions bind to ferrozine, forming a complex that absorbs light at 562 nm. In contrast, the sulfonamide **5b** remains stable at these conditions. Thus, substituting the sulfamoylcarbamate with a bioisosteric sulfonamide residue enhances the stability of compound **5b** against oxidative cleavage in the presence of Fe^3+^, and increases its substrate selectivity, reaching a numeric value of IC_50_(LA)/IC_50_(AA) ratios as low as 0.001.

#### 2.2.2. Isoform Specificity of the Inhibitory Effect Induced by Compound **5b**

Finally, we explored whether the most substrate specific ALOX15 inhibitor (compound **5b**) also inhibits other ALOX isoforms (Table 2). The data obtained for human ALOX15 are similar to those obtained for the rabbit ortholog. For LA oxygenation, the half-inhibition concentration (IC_50_) was 0.007 ± 0.0005 μM, whereas the inhibitory activity against AA oxygenation was three orders of magnitude lower (1.28 ± 0.30 μM). The substrate specificity ratio (IC_50_(LA)/IC_50_(AA) was 0.005 (Table 2), and this value was one order of magnitude lower than the corresponding ratio reported previously for compound **1** on human ALOX15 [16]. These data indicate that compound **5b** exhibits a higher degree of substrate selectivity than compound **1**.

Testing compound **5b** on the purified mouse Alox15 showed a dramatic loss of inhibitory potency and the absence of any substrate selectivity. In fact, the IC_50_ values for LA and AA oxygenation were similar (34 μM). For human ALOX15B, compound **5b** did not exhibit any inhibitory activity up to an inhibitor concentration of 50 µM. Since **5b** is not a suitable inhibitor for human ALOX15, the calculation of the IC_50_(LA)/IC_50_(AA) ratio is not meaningful.

## 3. Materials and Methods

### 3.1. General

Most solvents and reagents were purchased from Acros (Geel, Belgium) or Sigma-Aldrich (Schnelldorf, Germany) and were used without further purification unless otherwise noted. The ^1^H and ^13^C NMR spectra were recorded using a 300 MHz Bruker DPX 300 spectrometer (Bruker Corporation, Billerica, MA, USA) in CDCl_3_, CD_3_OD, D_2_O, acetone-*d*_6_ or DMSO-*d*_6_. Chemical shifts in ^1^H NMR were referenced to the residual proton signal of CDCl_3_, CD_3_OD, D_2_O, acetone-*d*_6_ or DMSO-*d*_6_ at δ ^1^H = 7.26, 3.31, 4.79, 2.05 or 2.50 ppm, respectively. Chemical shifts in ^13^C NMR were referenced to the residual carbon signal of CDCl_3_, CD_3_OD, acetone-*d*_6_ or DMSO-*d*_6_ at δ ^13^C = 77.2, 49.0, 29.8 or 39.5 ppm, respectively. Chemical shifts are given in ppm, and spin–spin interaction constants in Hz. Column chromatography was carried out using silica gel (Macherey-Nagel, Düren, Germany, particle size 63–200 µm) as a stationary phase. Silica gel 60 F254 plates (Merck, Darmstadt, Germany) were used for thin-layer chromatography (TLC). Compounds were detected under UV light or after staining with an ethanolic (3%) solution of vanillin. Preparative HPLC was performed using a Knauer HPLC pump 64 system coupled with a differential refractometer and a UV-VIS detector (Knauer, Berlin, Germany) and equipped with a Luna C18(2), 100A column (75 × 30 mm, 5 µm particle size) (Phenomenex, Torrance, CA, USA). The isocratic elution system comprising ACN/THF/H_2_O/FA (63:7:30:0.1 *v*/*v*/*v*/*v*) at a flow rate of 25 mL/min was applied to achieve the best chromatographic performance. Mass spectra (EI) were recorded on an Agilent a 6890 N gas chromatograph coupled with a 5973 N mass spectral detector (Agilent, Santa Clara, CA, USA) using a HP-5MS column (30 m, coating thickness 0.5 µm, Agilent J&W, Palo Alto, CA, USA). An injector temperature of 300 °C, an ion source temperature of 230 °C and the electron energy of 70 eV were set. Helium was used as the carrier gas at a flow rate of 1 mL/min. Samples were eluted using the following temperature program: isothermally at 50 °C for 5 min, then from 50 to 320 °C at a rate of 30 °C/min, followed by an isothermal step at 320 °C for 10 min. Analytical HPLC was performed with an Agilent 1260 Infinity II LC-MS system (Agilent, Santa Clara, CA, USA) that was equipped with a quaternary gradient pump, an autosampler, a column oven, a variable-wavelength UV detector, and a single-quadrupole mass-selective detector LC/MSD iQ. The separation of the analytes was carried out at 30 °C on a YMC Triat RP C18 column (YMC Co. Ltd., Kyoto, Japan; 75 × 3.0 mm, 5 μm particle size). A gradient elution system composed of solvents A (H_2_O with 0.1% formic acid and 10 mM ammonium formate) and B (ACN with 0.1% formic acid) at a flow rate of 0.6 mL/min was used. Samples were eluted starting from 5% of system B at increasing system B concentrations followed by isocratic elution at 100% after 10 min. Mass spectrometric detection was performed using electrospray ionization (ESI). The ion source parameters were as follows: gas temperature 325 °C, gas flow rate 8 L/min, nebulizer pressure 40 psi, and capillary voltage 4000 V. For each ion, a dwell time of 333 ms and a fragmentor voltage of 150 V were applied. UV/vis-spectra were recorded using a Specord 50 Plus spectrophotometer (Analytik Jena GmbH Co KG, Jena, Germany). IR spectra were recorded with Lumex InfraLUM FT-08 FTIR spectrometer (Lumex, Sankt Petersburg, Russia) equipped with a Specac Quest accessory (Specac, Orpington, UK) with diamond crystal. Elemental analysis was performed with a Flash EA1112 analyzer (Thermo Finnigan Italia S.p.A., Rodano, Italy).

### 3.2. Chemical Synthesis

***2-Methoxy-5-(4-phenyl-1H-pyrrol-2-yl)aniline * (8).** To the suspension of NaH (43 mg, 1.78 mmol) in DMSO (600 µL), a solution of 1-(3-amino-4-methoxyphenyl)ethan-1-one **7** (200 mg, 1.21 mmol) in DMSO (200 μL) was added dropwise over 3 min. To the resulting mixture, 3-phenyl-2*H*-azirine **6** (210 mg, 1.78 mmol) was added. The reaction was stirred at rt overnight, then quenched with brine; organic compounds were extracted with EtOAc (3 × 1 mL), and the combined extracts were dried over Na_2_SO_4_ and evaporated. The crude product was purified on silica gel using DCM as the eluent to yield compound **8**: 73% (235 mg). TLC: R*_f_* 0.55 (DCM/EtOAc 9:1, *v*/*v*). ^1^H NMR (300 MHz, DMSO-*d*_6_) δ 11.15 (s, 1H), 7.62–7.51 (m, 2H), 7.31 (t, *J* = 7.5 Hz, 2H), 7.24–7.18 (m, 1H), 7.11 (t, *J* = 7.2 Hz, 1H), 6.96 (d, *J* = 1.4 Hz, 1H), 6.92–6.77 (m, 2H), 6.68–6.59 (m, 1H), 4.69 (s, 2H), 3.78 (s, 3H). ^13^C NMR (75 MHz, DMSO-*d*_6_) δ 145.2, 137.6, 136.0, 133.2, 128.5, 125.9, 124.8, 124.3, 124.2, 115.3, 112.0, 110.8, 109.8, 101.4, 55.3. MS (EI, 70 eV) *m*/*z* (%): 264 (100), 249 (92), 110 (16), 204 (12), 132 (10).

***Octyl (N-(2-methoxy-5-(4-phenyl-1H-pyrrol-2-yl)phenyl)sulfamoyl)carbamate* (3).** Chlorosulfonyl isocyanate (35.50 μL, 0.41 mmol) was added to a solution of octan-1-ol **9** (58.60 μL, 0.37 mmol) in dry DCM (2 mL). The mixture was first stirred for 15 min at rt, then TEA (62 μL, 0.41 mmol) was added and the mixture was additionally stirred for 15 min. Finally, 2-methoxy-5-(4-phenyl-1*H*-pyrrol-2-yl)aniline (**8**) (98 mg, 0.17 mmol) was added and the resulting mixture was stirred at rt for 3 h and quenched with H_2_O (1.5 mL). The organic layer was separated, dried with Na_2_SO_4_ and concentrated *in vacuo*. The raw product was purified on silica gel (Pet/THF 2:1, *v*/*v*) followed by preparative RP-HPLC to yield compound **3**: 54% (43 mg). TLC: R*_f_* 0.56 (Pet/THF 2:1, *v*/*v*). Analytical HPLC: R*_t_* 9.65 min (235 nm), purity 99.0%. UV/vis λ_max_ 240, 295 nm. ^1^H NMR (300 MHz, CD_3_OD) δ 7.70 (d, *J* = 2.0 Hz, 1H), 7.60–7.54 (m, 2H), 7.39 (dd, *J* = 8.5, 2.0 Hz, 1H), 7.34–7.26 (m, 2H), 7.18–7.07 (m, 2H), 7.01 (d, *J* = 8.6 Hz, 1H), 6.76 (d, *J* = 1.5 Hz, 1H), 4.10 (t, *J* = 6.9 Hz, 2H), 3.87 (s, 3H), 1.63–1.47 (m, 2H), 1.33–1.16 (m, 10H), 0.88 (t, *J* = 6.9 Hz, 3H). ^13^C NMR (75 MHz, CD_3_OD) δ 190.3, 150.3, 137.6, 133.7, 129.5, 128.0, 127.5, 126.9, 126.1, 125.7, 122.0, 118.6, 116.5, 112.7, 103.6, 67.4, 56.6, 32.9, 30.3, 29.7, 26.8, 23.7, 14.4. MS (ESI): [M + H]^+^ 500.

***6-Bromo-N,N-dimethylhexanamide* (11).** Oxalyl chloride (774 μL, 9.00 mmol) was dissolved in 1 mL of dry DCM and the solution was added dropwise to a solution of 6-bromohexanoic acid (**10**) (1.17 g, 6.00 mmol) pre-cooled to 0 °C in a mixture of dry DCM (15 mL) and DMF (10 μL). The resulting mixture was warmed up to rt and stirred for 4 h, evaporated, and used in the next stage without further purification. Crude 6-bromohexanoyl chloride (R*_f_* 0.83, Pet/EA 7:3, *v*/*v*) was dissolved in 1 mL of dry DCM and the solution was added dropwise to a solution of dimethylamine hydrochloride (0.73 g, 9.00 mmol) and DIPEA (3.13 mL, 18.00 mmol) pre-cooled to 0 °C in dry DCM (10 mL). The solution was stirred for 20 min at 0 °C and additionally for 3 h at rt, then quenched with H_2_O, and the organic layer was separated. The aqueous layer was extracted with DCM (2 × 10 mL), and the combined organic layers were dried by passing through Na_2_SO_4_ and evaporated. Yield of compound **11**: 96% (1.27 g). TLC: R*_f_* 0.53 (Pet/DCM/TEA 1:5:0.1, *v*/*v*/*v*). ^1^H NMR (300 MHz, CDCl_3_) δ 3.40 (t, *J* = 6.8 Hz, 2H), 2.99 (s, 3H), 2.93 (s, 3H), 2.31 (t, *J* = 7.4 Hz, 2H), 1.94–1.82 (m, 2H), 1.72–1.59 (m, 2H), 1.54–1.43 (m, 2H). ^13^C NMR (75 MHz, CDCl_3_) δ 172.8, 37.4, 35.5, 33.9, 33.2, 32.7, 28.1, 24.3. MS (EI, 70 eV) *m*/*z* (%): 87 (100), 72 (46), 45 (29), 142 (18).

***6-(Dimethylamino)-6-oxohexyl benzoate* (12).** Benzoic acid (1.65 g, 13.50 mmol), potassium carbonate (1.87 g, 13.50 mmol), potassium iodide (20 mg), 6-bromo-*N,N*-dimethylhexanamide (**11**) (1.00 g, 4.50 mmol) and THF (30 mL) were mixed in a high-pressure glass bottle and stirred at 90 °C for 24 h. The resulting mixture was cooled down, quenched with H_2_O (30 mL), and extracted with CHCl_3_ (3 × 20 mL). The combined organic layers were washed with brine (40 mL), dried by passing through Na_2_SO_4_, and evaporated. Yield of compound **12**: 89% (1.05 g). TLC: R*_f_* 0.85 (CHCl_3_/MeOH 9:1, *v*/*v*). ^1^H NMR (300 MHz, CDCl_3_) δ 8.06–7.97 (m, 2H), 7.58–7.48 (m, 1H), 7.47–7.37 (m, 2H), 4.31 (t, *J* = 6.6 Hz, 2H), 2.98 (s, 3H), 2.92 (s, 3H), 2.38–2.25 (m, 2H), 1.93–1.58 (m, 6H). ^13^C NMR (75 MHz, CDCl_3_) δ 172.9, 166.8, 133.0, 130.5, 129.7, 128.5, 65.0, 37.4, 35.5, 33.3, 28.8, 26.1, 24.9. MS (EI, 70 eV) *m*/*z* (%): 87 (100), 105 (80), 77 (36), 72 (33), 45 (23).

***6-(Dimethylamino)hexan-1-ol* (13).** Lithium aluminum hydride (0.27 g, 7.00 mmol) was added portion-wise to a solution of 6-(dimethylamino)-6-oxohexyl benzoate (**12**) (0.92 g, 3.50 mmol) pre-cooled to −20 °C in dry THF (20 mL). The reaction was stirred at rt for 3 h, then cooled to −20 °C, quenched with ice-cold 45% aq. NaOH (100 mL) and extracted with Et_2_O (2 × 40 mL). The combined extracts were washed with brine, dried over Na_2_SO_4_, and the solvent was removed under reduced pressure. The raw product was purified on silica gel using a gradient elution (THF/TEA, 6:0 to 6:1, *v*/*v*). Yield of compound **13**: 65% (0.33 g). TLC: R*_f_* 0.66 (THF/TEA 6:1, *v*/*v*). ^1^H NMR (300 MHz, Acetone-*d*_6_) δ 3.51 (t, *J* = 6.4 Hz, 2H), 2.23–2.16 (m, 2H), 2.12 (s, 6H), 1.55–1.28 (m, 8H). ^13^C NMR (75 MHz, CDCl_3_) δ 62.3, 59.1, 44.7, 32.6, 26.9, 26.6, 25.5. MS (EI, 70 eV) *m*/*z* (%): 58 (100), 42 (6), 145 (3).

***6-(Dimethylamino)hexyl (N-(5-(1H-indol-2-yl)-2-methoxyphenyl)sulfamoyl)carbamate* (4a).** Chlorosulfonyl isocyanate (20 μL, 0.23 mmol) was added to a solution of 6-(dimethylamino)hexan-1-ol **13** (34 mg, 0.23 mmol) in dry DCM (2 mL). The mixture was stirred at rt for 15 min, then TEA (58 μL, 0.42 mmol) was added, and the mixture was additionally stirred for 15 min. Finally, 5-(1*H*-indol-2-yl)-2-methoxyaniline (**16**) (50 mg, 0.21 mmol) was added, the resulting mixture was stirred for 3 h at rt and concentrated *in vacuo*. The raw product was purified on silica gel (DCM/MeOH/TEA 8:1:1, *v*/*v*/*v*) followed by preparative RP-HPLC to yield compound **4a**: 69% (71 mg). TLC: R*_f_* 0.1 (THF/TEA 6:1, *v*/*v*). Analytical HPLC: R*_t_* 8.67 min (235 nm), purity 98.5%. UV/vis λ_max_ 234, 308. ^1^H NMR (300 MHz, CD_3_OD) δ 7.92 (d, *J* = 2.2 Hz, 1H), 7.52–7.45 (m, 1H), 7.41–7.34 (m, 2H), 7.10–7.02 (m, 1H), 7.01–6.93 (m, 2H), 3.93 (t, *J* = 6.1 Hz, 2H), 3.90 (s, 3H), 2.63–2.53 (m, 2H), 2.45 (s, 6H), 1.56–1.14 (m, 8H). ^13^C NMR (75 MHz, CDCl_3_) δ 160.6, 147.3, 138.6, 136.8, 136.7, 129.5, 125.7, 121.7, 120.2, 120.0, 115.7, 112.1, 111.0, 110.8, 98.4, 62.6, 59.2, 55.7, 44.7, 32.6, 26.8, 26.6, 25.5. MS (ESI): [M + H]^+^ 489.

***1-Trimethylsilyl triethylene glycol (mono-TMS-TEG)* (15).** HMDS (0.42 mL, 2 mmol) and BSTFA (20 μL) were added to a solution of triethylene glycol **14** (1.20 g, 8 mmol) in dry THF (10 mL) under the Ar atmosphere. The solution was stirred at reflux temperature for 21 h. The resulting mixture was evaporated and the crude product was purified on silica gel using CHCl_3_ as the eluent. Yield of compound **15**: 58% (0.26 g). TLC: R*_f_* 0.56 (CHCl_3_/MeOH 19:1, *v*/*v*). ^1^H NMR (300 MHz, CDCl_3_) δ 3.82–3.50 (m, 12H), 0.18–0.08 (m, 9H). ^13^C NMR (75 MHz, CDCl_3_) δ 72.7, 70.8, 70.5, 62.1, 62.0, 61.9, −0.3. MS (EI, 70 eV) *m*/*z* (%): 73 (100), 45 (85), 75 (66), 116 (45).

***2-(2-(2-Hydroxyethoxy)ethoxy)ethyl (N-(5-(1H-indol-2-yl)-2-methoxyphenyl)sulfamoyl)carbamate* (4b).** A solution of TEA (29 μL, 0.2 mmol) in dry DCM (200 μL) was added dropwise to a solution of chlorosulfonyl isocyanate (9.1 μL, 0.1 mmol) in dry DCM (200 μL) cooled to −10 °C. The mixture was stirred for 15 min, then a solution of mono-TMS-TEG **15** (0.1 mmol) in 200 μL dry DCM was added, and the mixture was additionally stirred for 15 min. Finally, 5-(1*H*-indol-2-yl)-2-methoxyaniline **16** (25 mg, 0.1 mmol) was added and the resulting mixture was stirred for 15 min at −10 °C and 3 h at rt, concentrated in vacuo, and purified on silica gel using CHCl_3_ as a solvent followed by preparative RP-HPLC. Yield of **4b**: 30% (16 mg). TLC: R*_f_* 0.25 (CHCl_3_/MeOH 19:1, *v*/*v*). Analytical HPLC: R*_t_* 10.88 min (235 nm), purity 98.7%. UV/vis λ_max_ 239, 311. ^1^H NMR (300 MHz, DMSO-*d*_6_) δ 11.30 (s, 1H), 7.75 (d, *J* = 2.2 Hz, 1H), 7.64 (dd, *J* = 8.6, 2.2 Hz, 1H), 7.49 (d, *J* = 7.7 Hz, 1H), 7.40–7.34 (m, 1H), 7.13 (d, *J* = 8.6 Hz, 1H), 7.09–7.02 (m, 1H), 7.00–6.93 (m, 1H), 6.74–6.69 (m, 1H), 4.22–4.13 (m, 2H), 3.82 (s, 3H), 3.64–3.54 (m, 2H), 3.54–3.40 (m, 6H), 3.42–3.33 (m, 2H). ^13^C NMR (75 MHz, DMSO-*d*_6_) δ 152.2, 151.3, 137.2, 137.0, 128.7, 125.5, 125.0, 123.5, 122.4, 121.2, 119.7, 119.3, 112.2, 111.2, 97. 8, 72.3, 69.7, 68.2, 64.8, 60.2, 56.0. MS (ESI): [M + H]^+^ 494.

***Octyl methanesulfonate* (18).** Mesyl chloride (0.71 mL, 9.21 mmol) was added dropwise to pre-cooled to 0 °C solution of octan-1-ol (**9**) (1.00 g, 7.68 mmol) and TEA (1.60 mL, 11.52 mmol) in dry DCM (10 mL). The reaction was stirred at 0 °C for 1.5 h and additionally at rt for 3 h, then quenched with H_2_O (5 mL), and the organic layer was separated, washed with 1M HCl (5 mL), saturated aq. NaHCO_3_ (5 mL), brine (5 mL), dried over Na_2_SO_4_ and evaporated. Yield of compound **18**: 98% (1.58 g). TLC: R*_f_* 0.75 (Pet/EA 7:3, *v*/*v*). ^1^H NMR (300 MHz, CDCl_3_) δ 4.21 (t, *J* = 6.6 Hz, 2H), 2.99 (s, 3H), 1.79–1.67 (m, 2H), 1.45–1.20 (m, 10H), 0.93–0.81 (m, 3H). ^13^C NMR (75 MHz, CDCl_3_) δ 97.6, 70.4, 37.5, 31.8, 29.2, 29.1, 25.5, 22.7, 14.2. MS (EI, 70 eV) *m*/*z* (%): 84 (100), 56 (99), 70 (98), 79 (72), 97 (59).

***1-Iodooctane* (19).** To a solution of compound **18** (0.52 g, 2.50 mmol) in acetone (15 mL), NaI·2H_2_O (1.86 g, 10.0 mmol) was added. The resulting mixture was stirred at reflux temperature for 16 h before it was concentrated in vacuo. The residue was dissolved in water (10 mL), extracted with hexanes (2 × 10 mL), dried under Na_2_SO_4_ and evaporated. Yield of compound **19**: 93% (0.56 g). TLC: R*_f_* 0.95 (Pet). ^1^H NMR (300 MHz, CDCl_3_) δ 3.19 (t, *J* = 7.1 Hz, 2H), 1.89–1.74 (m, 2H), 1.44–1.22 (m, 10H), 0.88 (t, *J* = 6.6 Hz, 3H). ^13^C NMR (75 MHz, CDCl_3_) δ 33.7, 31.9, 30.7, 29.2, 28.7, 22.8, 14.2, 7.5. MS (EI, 70 eV) *m*/*z* (%): 71 (100), 57 (50), 155 (29), 41 (29), 240 (20).

***Sodium octane-1-sulfonate* (20).** The solution of Na_2_SO_3_ (0.39 g, 3.12 mmol) in water (5 mL) was added to the solution of 1-iodooctane (**19**) (0.5 g, 2.08 mmol) in MeOH (4 mL). The resulting mixture was first stirred at rt for 30 min, and then additionally under reflux temperature for 16 h. After the reaction was complete, the mixture was cooled down, quenched with MeOH (10 mL), and the precipitate was filtered off. The filtrate was evaporated, and the residue was washed with a small amount of cold acetone. Yield of compound **20**: 88% (0.40 g). ^1^H NMR (300 MHz, D_2_O) δ 3.05–2.95 (m, 2H), 1.89–1.76 (m, 2H), 1.57–1.34 (m, 10H), 0.96 (t, *J* = 6.4 Hz, 3H). ^13^C NMR (75 MHz, D_2_O) δ 51.1, 31.0, 28.2, 27.7, 23.9, 22.0, 13.4. IR (ATR, diamond), cm^−1^: 3536, 2920, 2858, 1459, 1050, 609.

***Octane-1-sulfonyl chloride* (21).** The solution of oxalyl chloride (103 μL, 1.2 mmol) in 0.4 mL of dry DCM was added dropwise to the mixture of sodium octane-1-sulfonate (**20**) (0.13 g, 0.6 mmol), dry DCM (1 mL) and DMF (20 μL) pre-cooled to −15 °C. The mixture was stirred at −15 °C for 10 min and at rt for 4 h. The precipitate was filtered off, and the filtrate was evaporated. The crude compound **21** (0.12 g) was used in the next stage without purification and characterization. TLC: R*_f_* 0.9 (Pet/EA 7:3, *v*/*v*).

#### General Procedure for the Preparation of Compounds **5a**,**b**

To a suspension of 5-(1*H*-indol-2-yl)-2-methoxyaniline (**16**) (119 mg, 0.5 mmol) in 2 mL of dry DCM, triethylamine (104 μL, 0.75 mmol) was added. To the resulting mixture cooled down to 0 °C, a solution of corresponding sulfonyl chloride **17** or **21** (0.6 mmol) in 0.5 mL of dry DCM was added dropwise. The mixture was stirred at rt for 5 h, quenched with 1 M HCl (1 mL), and the organic layer was separated. The aqueous layer was extracted with DCM (2 × 1 mL), and combined organic extracts were washed with a sat. solution of NaHCO_3_ (2 mL) and brine (2 mL), dried by passing through Na_2_SO_4_, and evaporated. The raw product was purified on silica gel using DCM as the eluent.

***N-(5-(1H-indol-2-yl)-2-methoxyphenyl)-4-pentylbenzenesulfonamide* (5a):** Yield: 56%. TLC: R*_f_* 0.71 (DCM). Analytical HPLC: R*_t_* 14.00 min (235 nm), purity 98.1%. UV/vis λ_max_ 235, 311 nm. ^1^H NMR (300 MHz, Acetone-*d*_6_) δ 10.70 (s, 1H), 7.93 (d, *J* = 2.2 Hz, 1H), 7.76–7.67 (m, 2H), 7.61–7.51 (m, 2H), 7.40 (d, *J* = 8.0 Hz, 1H), 7.38–7.29 (m, 2H), 7.13–6.92 (m, 3H), 6.75 (d, *J* = 2.2 Hz, 1H), 3.63 (s, 3H), 2.68–2.59 (m, 2H), 1.64–1.53 (m, 2H), 1.35–1.23 (m, 4H), 0.85 (d, *J* = 6.7 Hz, 3H). ^13^C NMR (75 MHz, DMSO-*d*_6_) δ 152.1, 147.5, 137.8, 137.2, 137.0, 128.7, 128.6, 126.8, 125.8, 124.8, 123.5, 122.8, 121.2, 119.7, 119.3, 112.2, 111.2, 97.6, 55.5, 34.8, 30.7, 30.2, 21.9, 13.9. MS (ESI): [M + H]^+^ 449. Elemental analysis calcd: C 69.62, H 6.29, N 6.25, S 7.15; found: 69.28, 6.28, 6.11, 7.27.

***N-(5-(1H-indol-2-yl)-2-methoxyphenyl)octane-1-sulfonamide* (5b):** Yield: 45%. TLC: R*_f_* 0.70 (DCM). Analytical HPLC: R*_t_* 13.92 min (235 nm), purity 98.6%. UV/vis λ_max_ 246, 311 nm. ^1^H NMR (300 MHz, CDCl_3_) δ 8.46 (s, 1H), 7.85 (d, *J* = 2.3 Hz, 1H), 7.60 (d, *J* = 7.7 Hz, 1H), 7.51–7.36 (m, 2H), 7.29–7.06 (m, 2H), 7.00–6.87 (m, 2H), 6.76 (d, *J* = 1.3 Hz, 1H), 3.91 (s, 3H), 3.15–3.01 (m, 2H), 1.90–1.73 (m, 2H), 1.42–1.11 (m, 10H), 0.86 (t, *J* = 6.6 Hz, 3H). ^13^C NMR (75 MHz, CDCl_3_) δ 148.6, 137.2, 136.9, 129.4, 127.0, 126.2, 122.4, 122.2, 120.6, 120.4, 116.5, 111.4, 111.1, 99.6, 56.1, 51.6, 31.8, 29.1, 29.0, 28.2, 23.5, 22.7, 14.2. MS (ESI): [M + H]^+^ 415. Elemental analysis calcd: C 66.64, H 7.29, N 6.76, S 7.73; found: 66.53, 7.37, 6.66, 8.01.

### 3.3. Logp and Solubility Measurements

To estimate the distribution coefficient for compounds **4a**–**b,** we used the spectrophotometric method. For this purpose, 1 μL (25 mM stock solution in DMSO) of the corresponding compound was dissolved in 1 mL of water and 1 mL of octan-1-ol was added. The mixture was shaken at rt for 10 min and the layers were separated. Log*p* values were determined as the ratio of the absorption intensities of the octanolic and aqueous phases at the corresponding λ_max_ (308 nm for **4a** and 311 nm for **4b**). The measurements were performed in triplicate. Acetamide (Log*p* −1.26) and 1,4-dichlorobenzene (Log*p* 3.37) were used as standards.

The solubility of compounds **1** and **4a**–**b** was estimated by the spectrophotometric method as the dependence of the UV-Vis absorbance on the corresponding analyte concentration. For this purpose, a zero sample and at least five non-zero samples covering the ranges of 1–100 µM of compounds **1** and **4a**–**b** (final concentration in a 1 mL cuvette) were plotted as the absorption intensities obtained at the corresponding λ_max_ of the analyte against the inhibitor concentrations in PBS. To assess the linearity of the curve, the line of best fit was determined by least square linear regression. Each data point represents the mean ± SEM of three independent activity measurements.

### 3.4. Enzyme Preparation

Wild-type rabbit and human ALOX15 were expressed as N-terminal His-tag fusion proteins in *E. coli* and purified as described previously [17,25]. mAlox15 and hALOX15B coding regions were inserted into the pET28b(+) expression vector between the SalI (N-terminus) and HindIII for mAlox15 [28] and between BamHI and HindIII restriction sites for hALOX15B. The proteins were expressed in *E. coli* BL21 (DE3) pLysS che cells (Evrogen, Moscow, Russia). The proteins were purified using affinity chromatography on Ni SepFast FF TED according to the protocol described previously [17,25]. Typically, an electrophoretic purity of >95% of the enzyme preparations was reached.

### 3.5. ALOX15 Activity Assay

The impact of the inhibitors on the rate of LA or AA oxygenation (25 μM final concentrations to obtain dose–response curves or varying substrate concentrations to establish the mechanism of inhibition) was assayed spectrophotometrically using a SPECORD 50 Plus spectrophotometer (Analytik Jena GmbH Co. KG, Jena, Germany) measuring the increase in conjugated diene formation (235 nm). The assay mixture was composed of a 0.1 M PBS at pH 7.4 and various concentrations of inhibitors. Both AA and LA were prepared as Na^+^-salt stock solutions in PBS (5 mM). No detergent was used. The compounds **3**, **4a**–**b**, **5a**–**b** were dissolved in DMSO, and serial dilutions were carried out so that from each dilution, 1 μL was applied for the measurement. Purified ALOX15 (2–5 μg) was pre-incubated with a testing compound for 1 min, and the reaction was started by the addition of the substrate. The linear part of the kinetic progress curve was evaluated. The activity of the solvent controls (1 µL DMSO) was set as 100%. Finally, the data were fitted to different inhibition models (competitive, mixed, pure noncompetitive and uncompetitive) using the software package GraphPad Prism 8, and the best fits were further evaluated.

## 4. Conclusions

Based on our experimental data, one can conclude that the replacement of the rigid indole core of the lead compound **1** by a 4-phenyl-1*H*-pyrrol-2-yl fragment resulted in a partial loss of the substrate-specific inhibitory potency for rabbit ALOX15. The replacement of the lipophilic hydrocarbon chain by a more hydrophilic moiety improved the water solubility of compounds **4a** and **4b**, but did not alter the substrate selectivity of LA-oxygenation. Unfortunately, the inhibitor potency of compounds **4a**–**b** was impaired by more than 1 order of magnitude. The bioisosteric replacement of the sulfamoylcarbamate moiety by a sulfonamide residue improved the degree of substrate selectivity of the novel inhibitors **5a** and **5b** for rabbit ALOX15 with IC_50_(LA)/IC_50_(AA) ratios of 0.003 (**5a**) and <0.001 (**5b**). The most effective inhibitor of human and rabbit ALOX15 was compound **5b**. It was highly potent (low IC_50_), strongly substrate-selective [IC_50_(LA)/IC_50_(AA) ratio of 0.001], and paralog specific (does not inhibit human ALOX15B). Moreover, this compound was less effective for mouse Alox15 (high ortholog specificity), and did not show any substrate selectivity for this enzyme, suggesting a different inhibitory mechanism for human and mouse ALOX15 orthologs.

## Figures and Tables

**Table 1:** Inhibitory activity of reference compound **1** and its analogues with LA and AA against recombinant rabbit ALOX15.

Compound	IC_50_(LA), μM	IC_50_(AA), μM	IC_50_(LA)/IC_50_(AA)
1 *	0.040 ± 0.001	2.06 ± 0.14	0.019
**3**	0.30 ± 0.02	4.40 ± 0.08	0.068
**4a**	2.29 ± 0.34	>50	<0.046
**4b**	1.33 ± 0.10	>50	<0.027
**5a**	0.036 ± 0.005	12.89 ± 0.70	0.003
**5b**	0.05 ± 0.01	>50	<0.001

* reference compound.

**Table 2:** Inhibitory activity of compound **5b** against recombinant ALOX15 isoforms.

Enzyme	IC_50_(LA), μM	IC_50_(AA), μM	IC_50_(LA)/IC_50_(AA)
mouse Alox15	34.44 ± 3.30	34.22 ± 4.14	~1
human ALOX15	0.007 ± 0.0005	1.28 ± 0.30	0.005
human ALOX15B	>50	>50	n/a *

* n/a—not applicable.

## Data Availability

The original contributions presented in this study are included in the article and Supplementary Materials. Further inquiries can be directed to the corresponding author.

## Supplementary Materials

The following supporting information can be downloaded at https://www.mdpi.com/article/10.3390/molecules31142544/s1, Figure S1: Inhibition of LA (A) and AA (B) oxygenation by rabbit ALOX15 in the presence of compound **3**; Figure S2: Molecular docking of compounds **1** and **3**; Figure S3: Inhibition of LA oxygenation by rabbit ALOX15 in the presence of compound **4b**; Figure S4: Log*p* (A) and solubility data (B) of compounds **1**, **4a** and **4b**; Figure S5. Inhibition of LA (A) oxygenation by rabbit ALOX15 in the presence of compounds **5a** and **5b**. Analytical data.
