# Supplementary material for: Structural Improvement of the Allosteric ALOX15 Inhibitor Octyl (N-(5-(1H-Indol-2-yl)-2-methoxyphenyl)sulfamoyl)carbamate
Source: Molecules. 2026 Jul 22;31(14):2544. doi: 10.3390/molecules31142544 (PMC13415832; doi:10.3390/molecules31142544)
Supplement: Supplementary file 1 [file molecules-31-02544-s001.zip › molecules-4422768-supplementary.pdf]

Supporting Information

Structural improvement of the allosteric ALOX15 inhibitor octyl (N-(5-(1*H*-indol-2-yl)-2-methoxyphenyl)sulfamoyl)carbamate

Authors: Viktor Gavrilyuk,[1] Vladislav Aksenov,[1] Kirill Petrov,[1] Dmitriy Bortnevsky,[1] Alexander Zhuravlev,[1] Alexey Golovanov,[1] Hartmut Kuhn,[2] Igor Ivanov*[1]

[1] Lomonosov Institute of Fine Chemical Technologies, MIREA - Russian Technological University, Vernadskogo pr. 86, 119571 Moscow, Russia; viktor_gavril@inbox.ru (V.G.); aksenov.v.v@edu.mirea.ru (V.A); alekszhur95@yandex.ru (A.Z.); ivanov_i@mirea.ru (I.I.)

[2] Institute of Biochemistry, Charite - University Medicine Berlin, Corporate member of Free University Berlin, Humboldt University Berlin and Berlin Institute of Health, Charitéplatz 1, D-10117 Berlin, Germany; hartmut.kuehn@charite.de

* Correspondence: ivanov_i@mirea.ru; Tel.: +7-(495)-246-05-55 (supl. 884)

Content

1. Figures 3

Figure S1. Inhibition of LA (A) and AA (B) oxygenation by rabbit ALOX15 in the presence of compound **3** 3

Figure S2. Molecular docking of compounds **1** and **3** 4

Figure S3. Inhibition of LA oxygenation by rabbit ALOX15 in the presence of compound **4b**. 5

Figure S4. Log*p* (A) and solubility data (B) of compounds **1**, **4a** and **4b**. 6

Figure S5. Inhibition of LA (A) oxygenation by rabbit ALOX15 in the presence of compounds **5a and 5b.** 7

2. Analytical Data 8

2-Methoxy-5-(4-phenyl-1H-pyrrol-2-yl)aniline (8) 8

Octyl (N-(2-methoxy-5-(4-phenyl-1H-pyrrol-2-yl)phenyl)sulfamoyl)carbamate (3) 10

6-Bromo-N,N-dimethylhexanamide (11) 12

6-(Dimethylamino)-6-oxohexyl benzoate (12) 14

6-(Dimethylamino)hexan-1-ol (13) 16

6-(Dimethylamino)hexyl (N-(5-(1H-indol-2-yl)-2-methoxyphenyl)sulfamoyl)-carbamate (4a) 18

1-Trimethylsilyl triethylene glycol (mono-TMS-TEG) (15) 20

2-(2-(2-Hydroxyethoxy)ethoxy)ethyl (N-(5-(1H-indol-2-yl)-2-methoxyphenyl)-sulfamoyl)carbamate (4b) 22

Octyl methanesulfonate (18) 24

1-Iodooctane (19) 26

Sodium octane-1-sulfonate (20) 28

N-(5-(1H-indol-2-yl)-2-methoxyphenyl)-4-pentylbenzenesulfonamide (5a) 30

N-(5-(1H-indol-2-yl)-2-methoxyphenyl)octane-1-sulfonamide (5b) 32

1. Figures

Figure S1. Inhibition of LA (A) and AA (B) oxygenation by rabbit ALOX15 in the presence of compound **3**

A

Michaelis-Menten Best-fit values	no inhibitor	1 µM **3**
k_{cat}	17.9±1.6	18.3±3.1
K_M	22.0±5.9	238.8±34.2

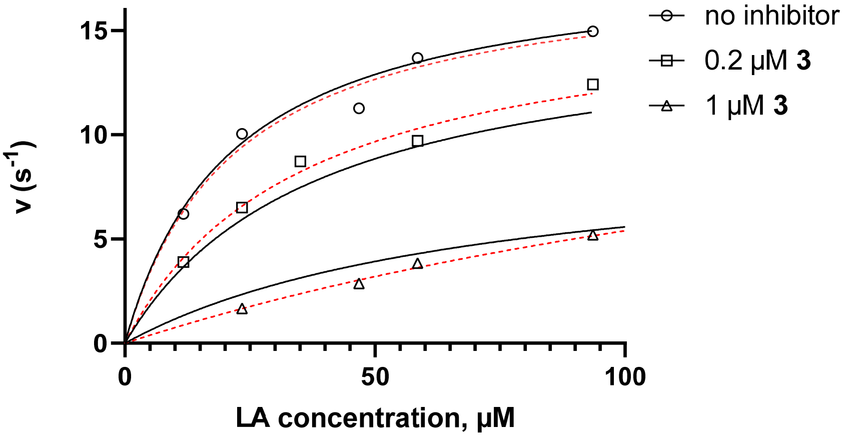

B

Michaelis-Menten Best-fit values	no inhibitor	5 µM **3**
k_{cat}	6.6±1.3	4.6±0.2
K_M	39.4±12.7	89.7±5.1

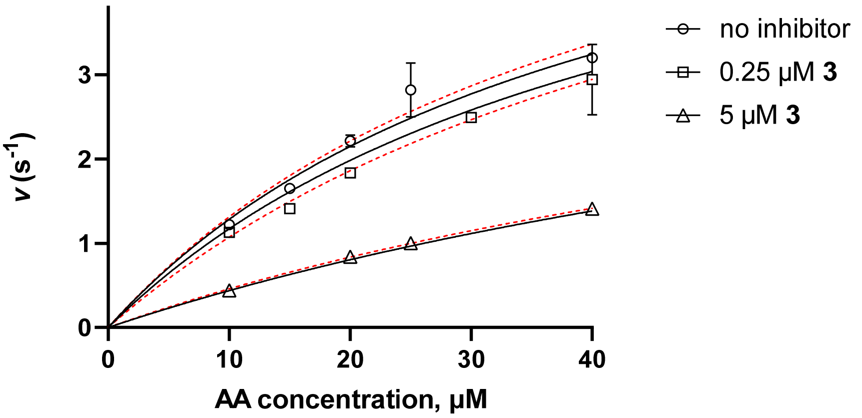

Figure S2. Molecular docking of compounds **1** and **3**

A

B

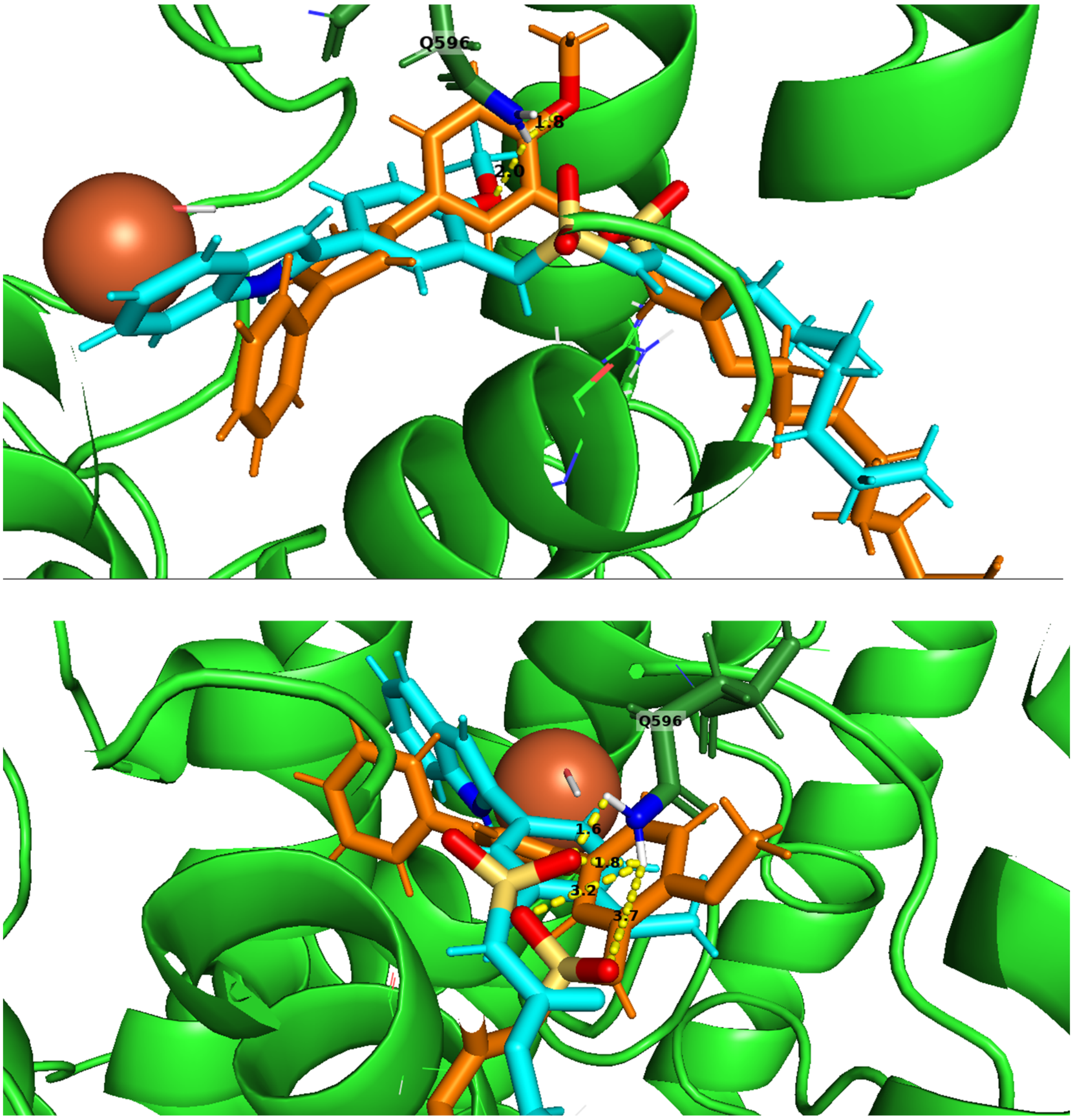

The position of compounds **1** (cyan) and **3** (orange) within the ALOX15 (PBD ID: 2P0M) binding pocket. A rotation of the molecule relative to the central phenyl ring is observed which locates the MeO-group somewhat closer to Q596 (**A**), while both the heterocyclic NH was more distanced from the Fe(III)-OH^- (distance not specified) and the SO_2 group was more distanced from the sidechain of Q596 (**B**).

Figure S3. Inhibition of LA oxygenation by rabbit ALOX15 in the presence of compound **4b**.

Michaelis-Menten Best-fit values	no inhibitor	5 μM **4b**
k_{cat}	12.9±1.1	2.7±0.2
K_M	29.1±6.1	22.22±5.4

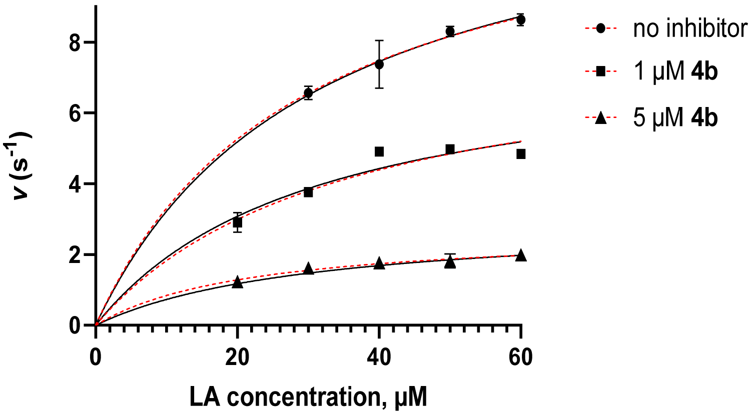

Figure S4. Log*p* (A) and solubility data (B) of compounds **1**, **4a** and **4b**.

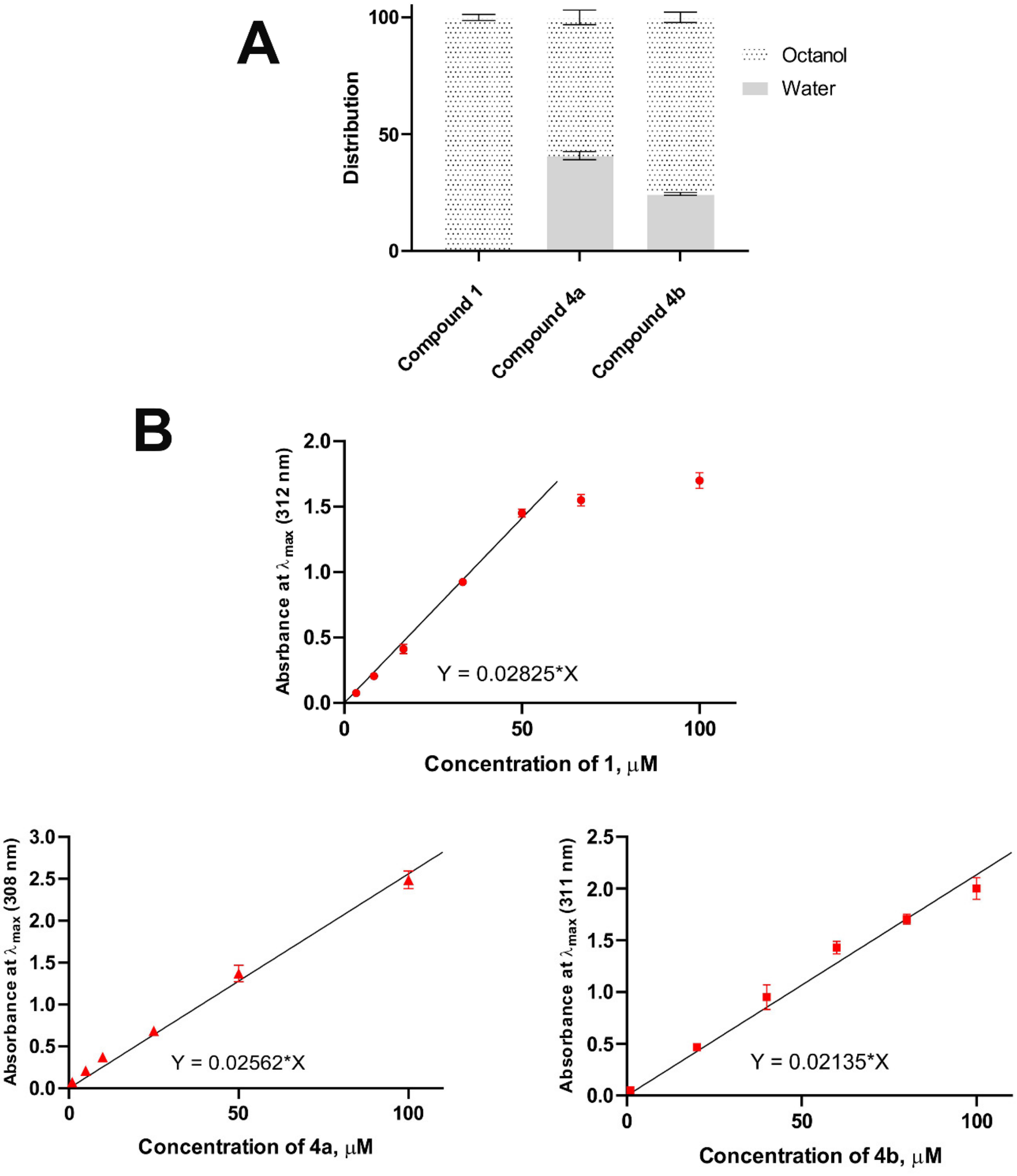

Figure S5. Inhibition of LA (A) oxygenation by rabbit ALOX15 in the presence of compound **5a and 5b.**

Michaelis-Menten Best-fit values	no inhibitor	50 nM **5a**
k_{cat}	18.2±3.0	5.46±1.1
K_M	34.8±12.5	24.9±10.2

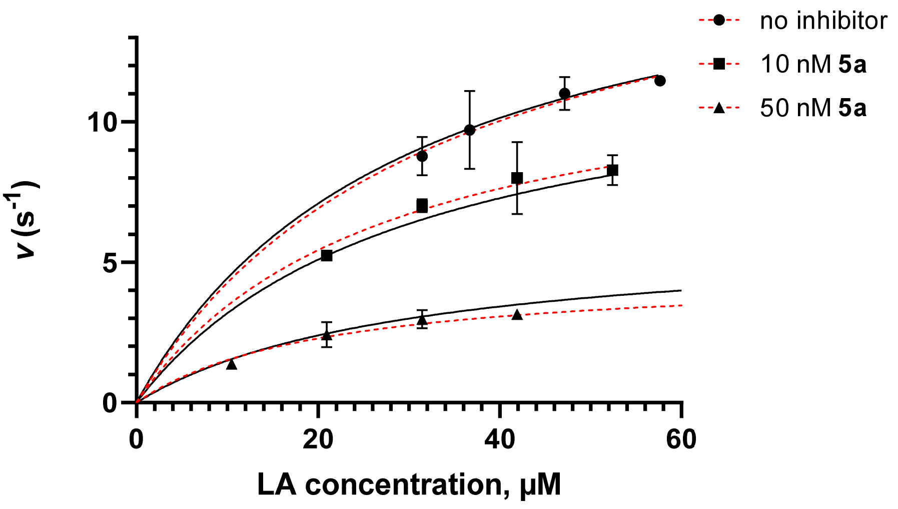

Michaelis-Menten Best-fit values	no inhibitor	100 nM **5b**
k_{cat}	14.2±2.1	3.4±0.4
K_M	40.1±14.1	35.9±9.0

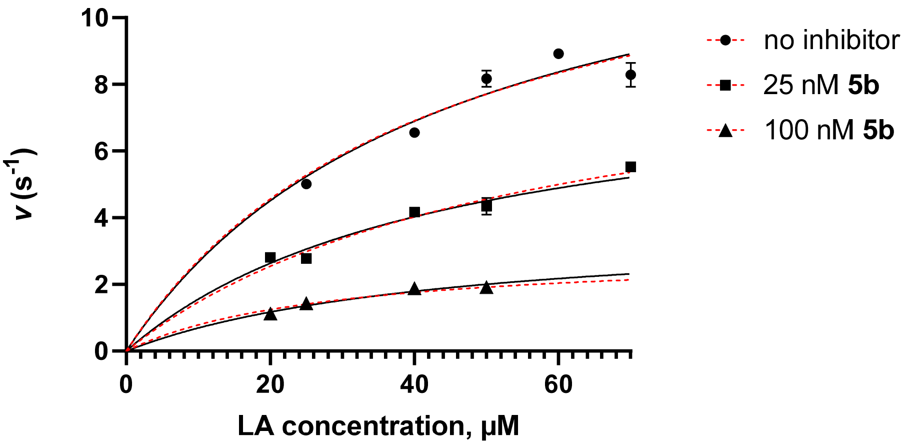

2. Analytical Data

2-Methoxy-5-(4-phenyl-1H-pyrrol-2-yl)aniline (8)

^{1}H NMR spectrum of compound **8**

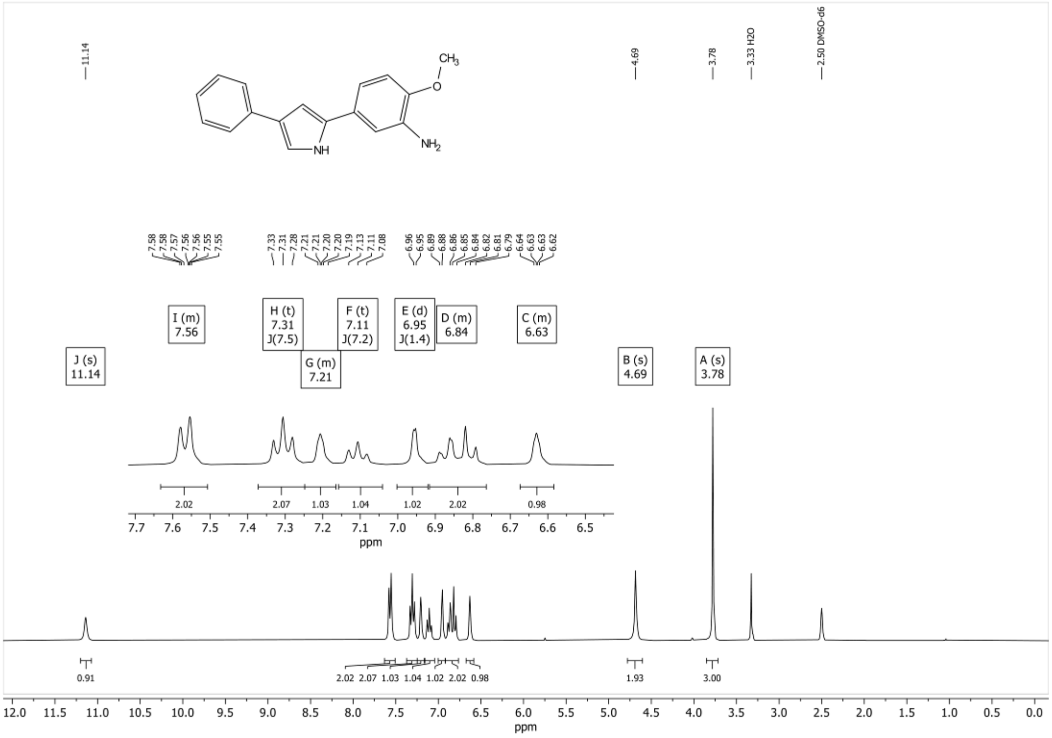

^{13}C NMR spectrum of compound **8**

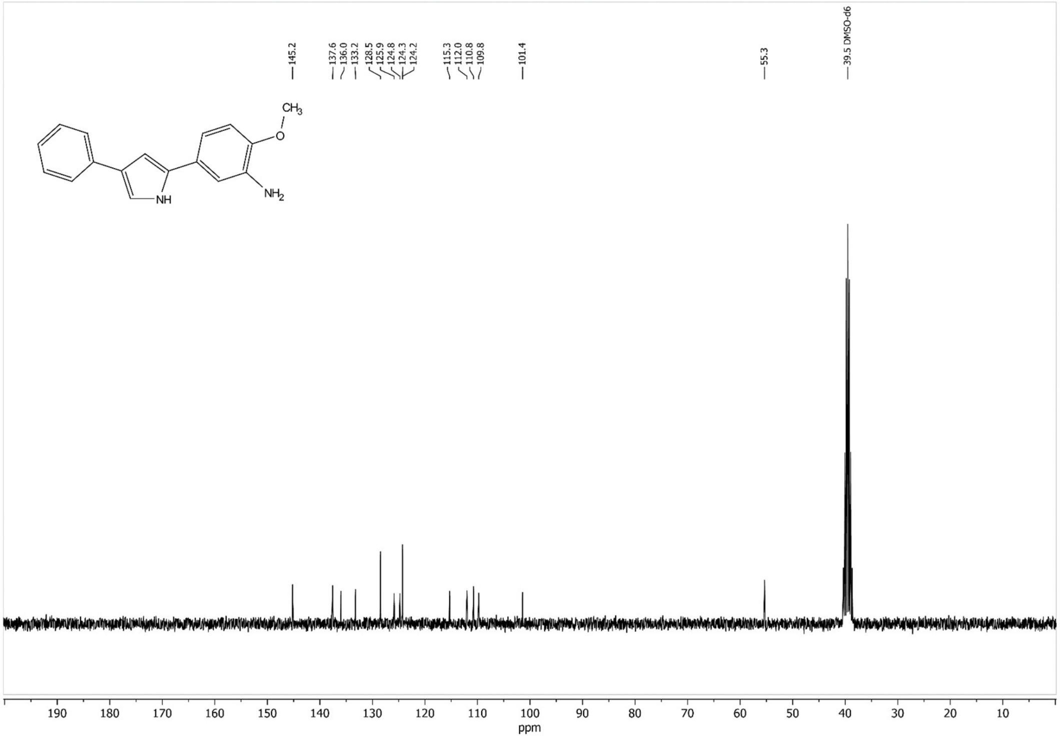

MS spectrum (EI) of compound **8**

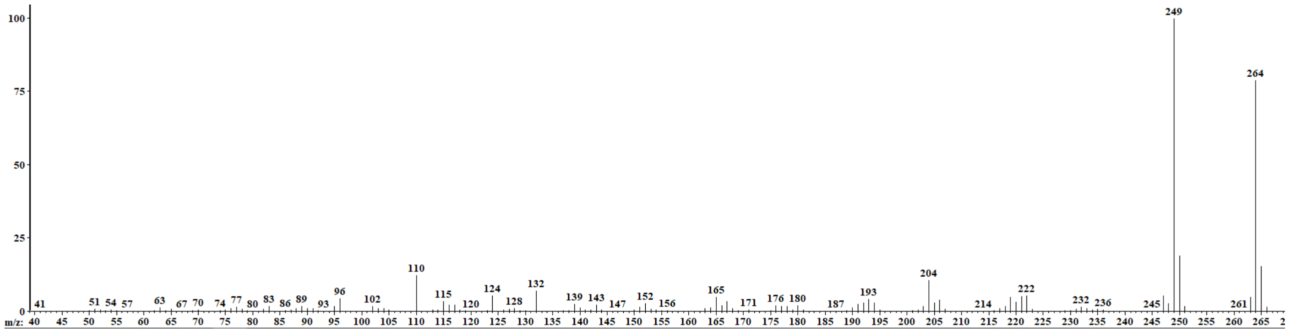

Octyl (N-(2-methoxy-5-(4-phenyl-1H-pyrrol-2-yl)phenyl)sulfamoyl)carbamate (3)

^{1}H NMR spectrum of compound **3**

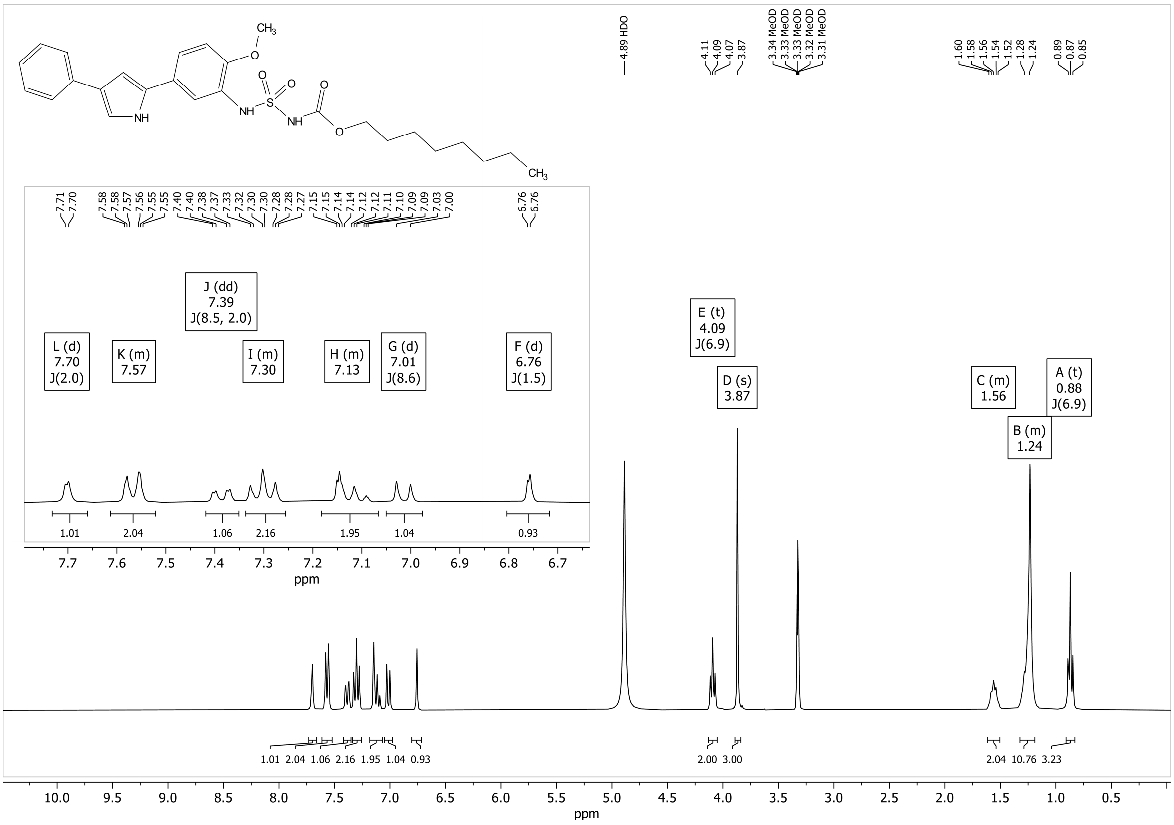

^{13}C NMR spectrum of compound **3**

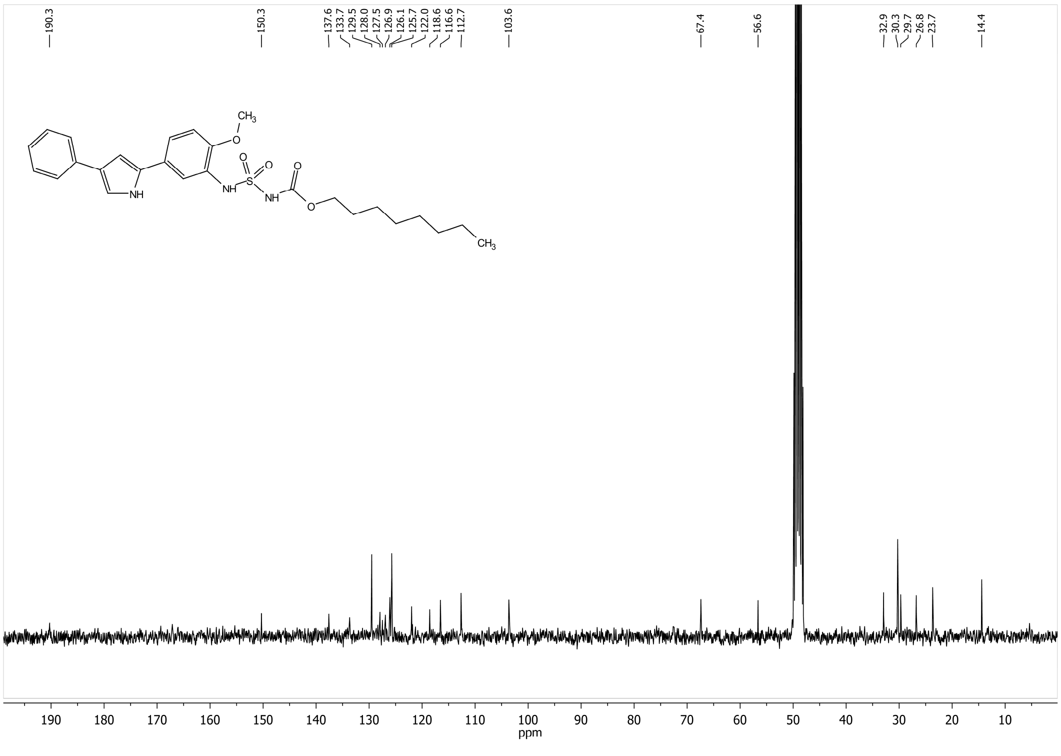

MS spectrum (ESI) of compound **3**

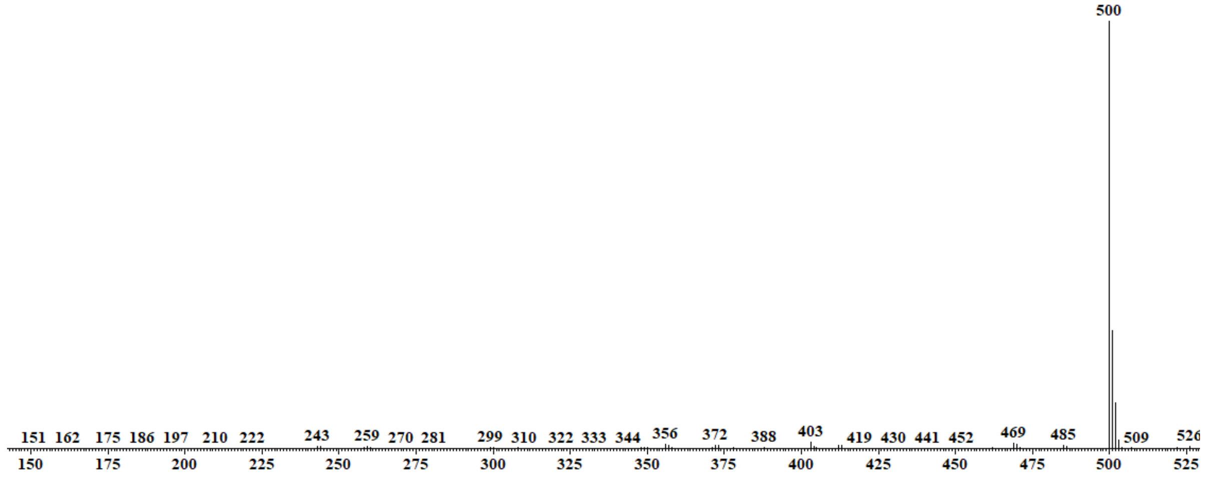

UV spectrum of compound **3**

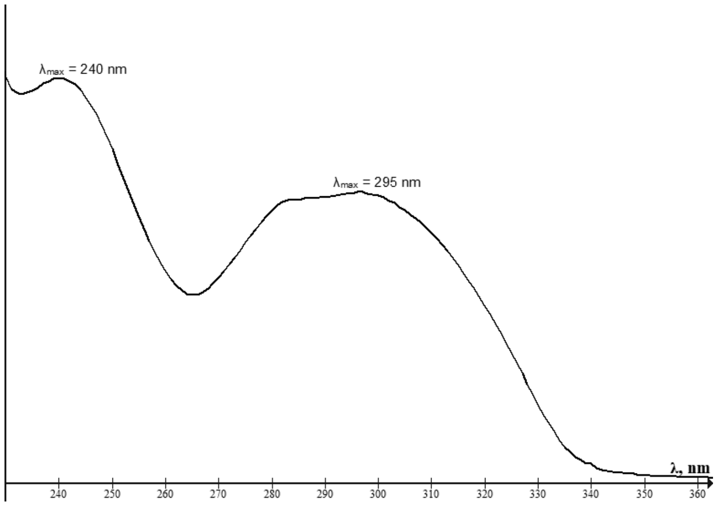

Analytical HPLC of compound 3:

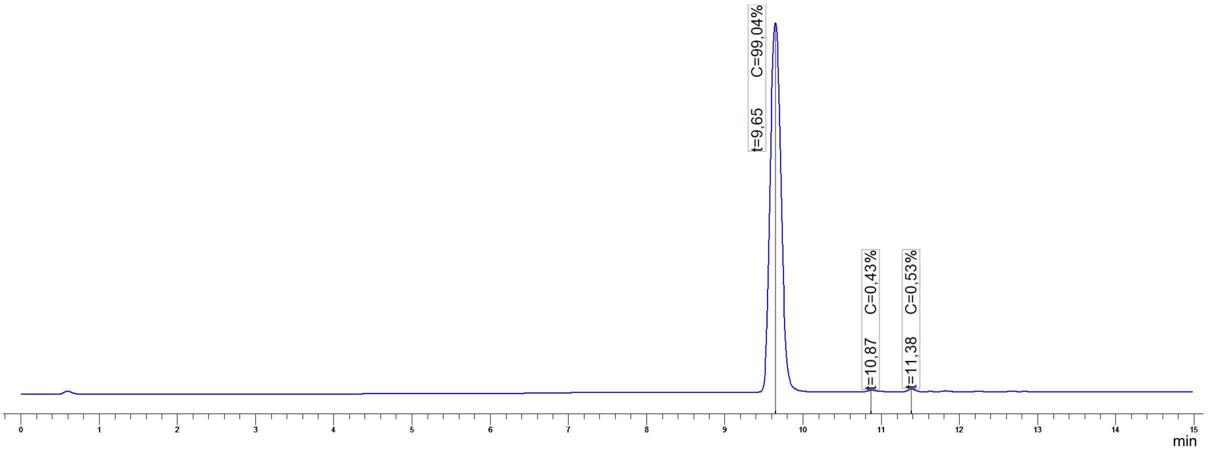

Gradient elution system composed of solvents A (H_2O with 0.1% formic acid and 10 mM ammonium formate) and B (ACN with 0.1% formic acid) at a flow rate of 0.6 mL/min was used. Mass spectrometric detection was performed using electrospray ionization (ESI).

6-Bromo-N,N-dimethylhexanamide (11)

^{1}H NMR spectrum of compound **11**

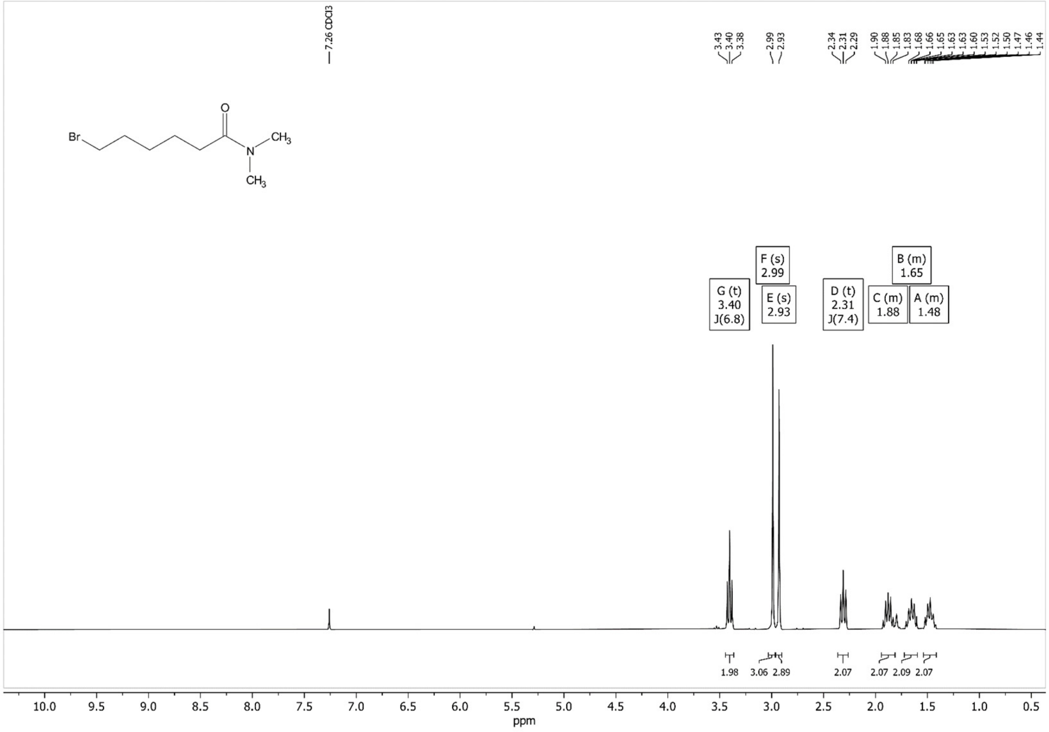

^{13}C NMR spectrum of compound **11**

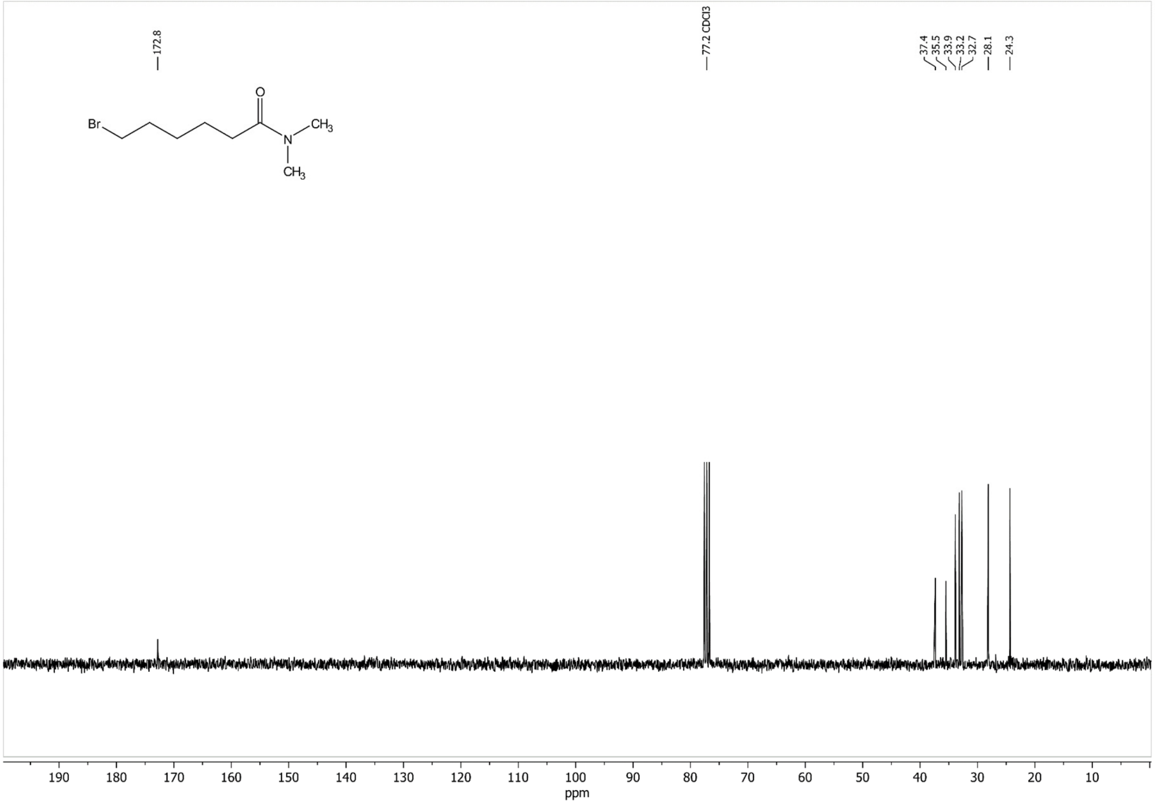

MS spectrum (EI) of compound **11**

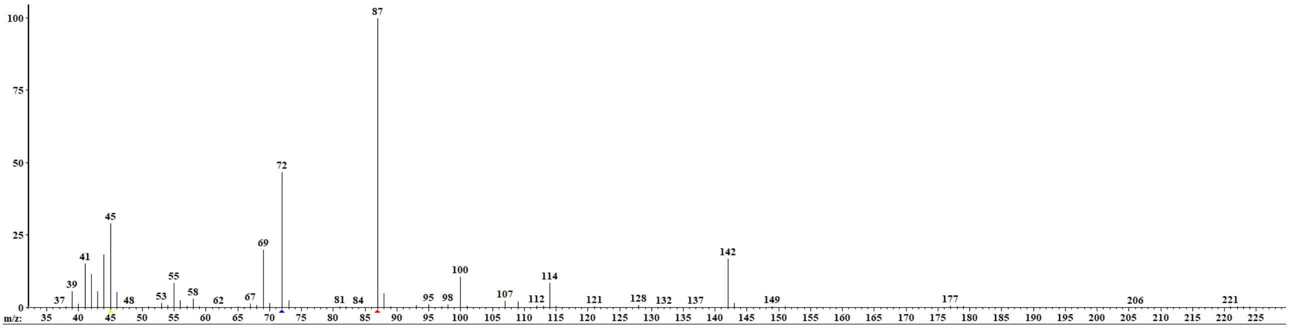

6-(Dimethylamino)-6-oxohexyl benzoate (12)

^{1}H NMR spectrum of compound **12**

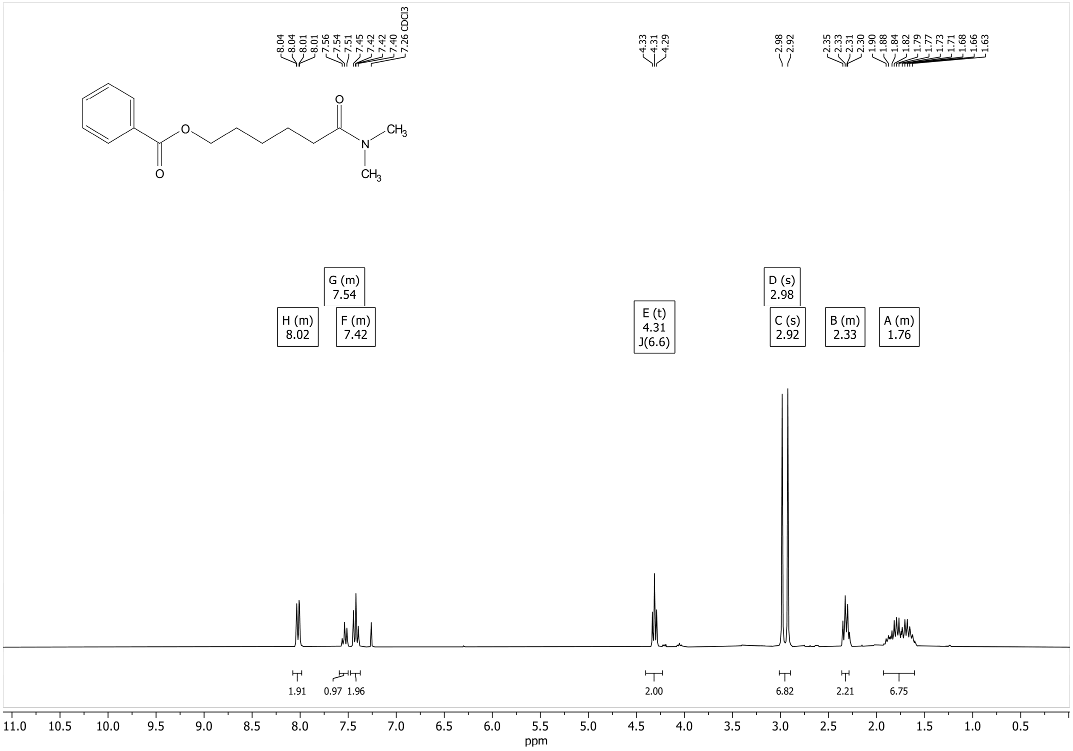

^{13}C NMR spectrum of compound **12**

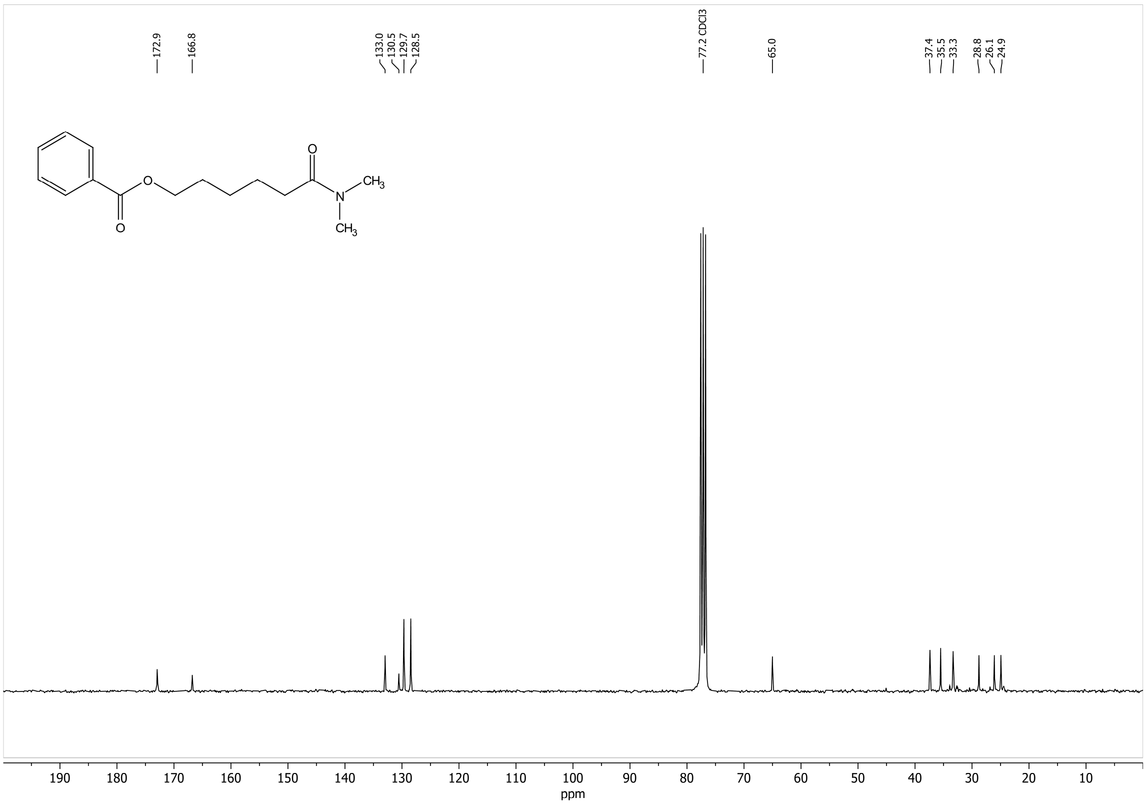

MS spectrum (EI) of compound **12**

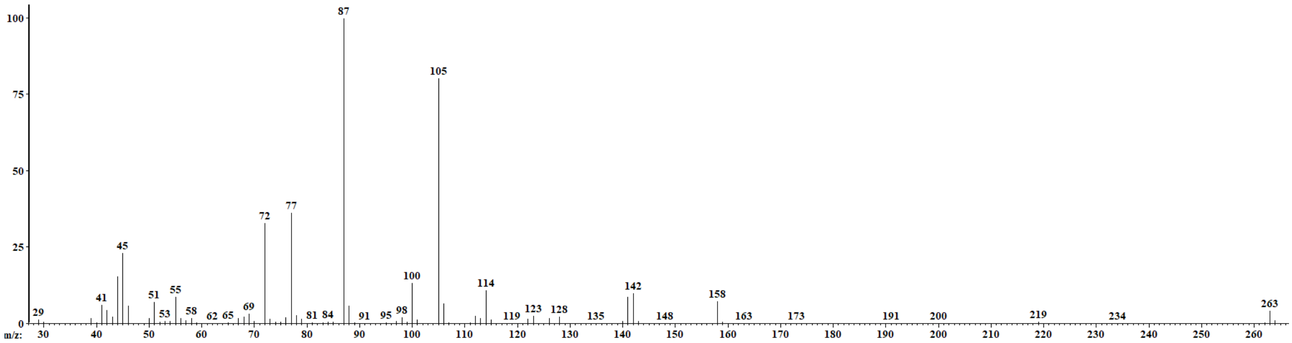

6-(Dimethylamino)hexan-1-ol (13)

^{1}H NMR spectrum of compound **13**

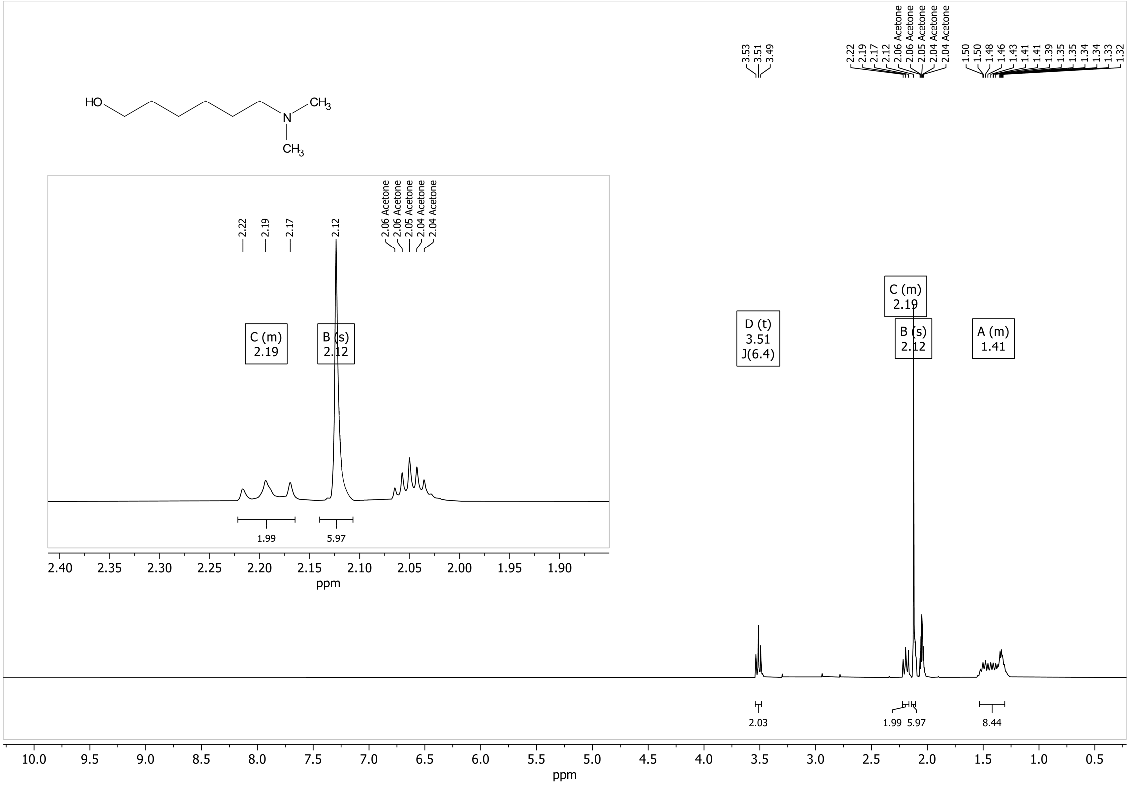

^{13}C NMR spectrum of compound **13**

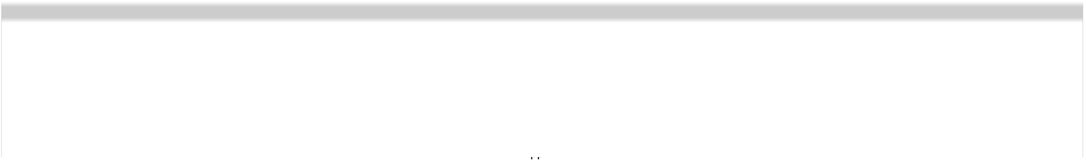

MS spectrum (EI) of compound **13**

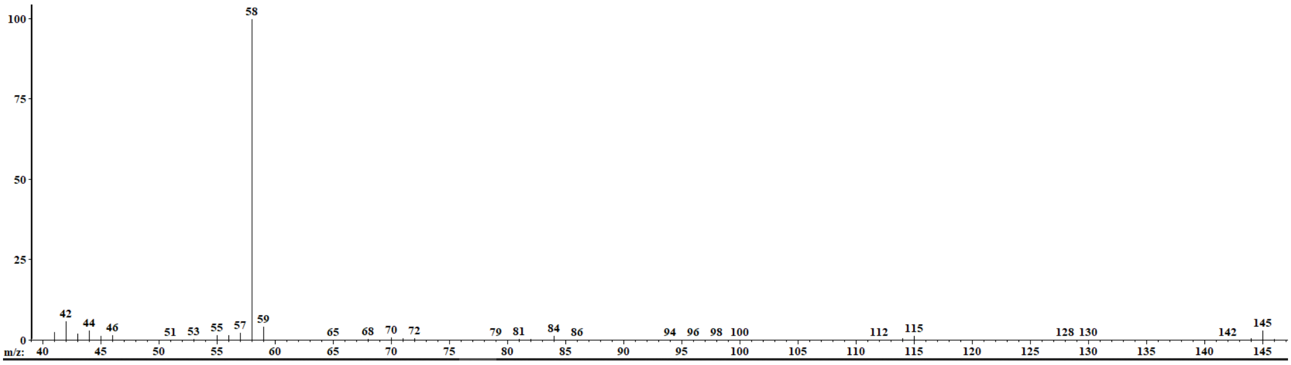

6-(Dimethylamino)hexyl (N-(5-(1H-indol-2-yl)-2-methoxyphenyl)sulfamoyl)-carbamate (4a)

^{1}H NMR spectrum of compound **4a**

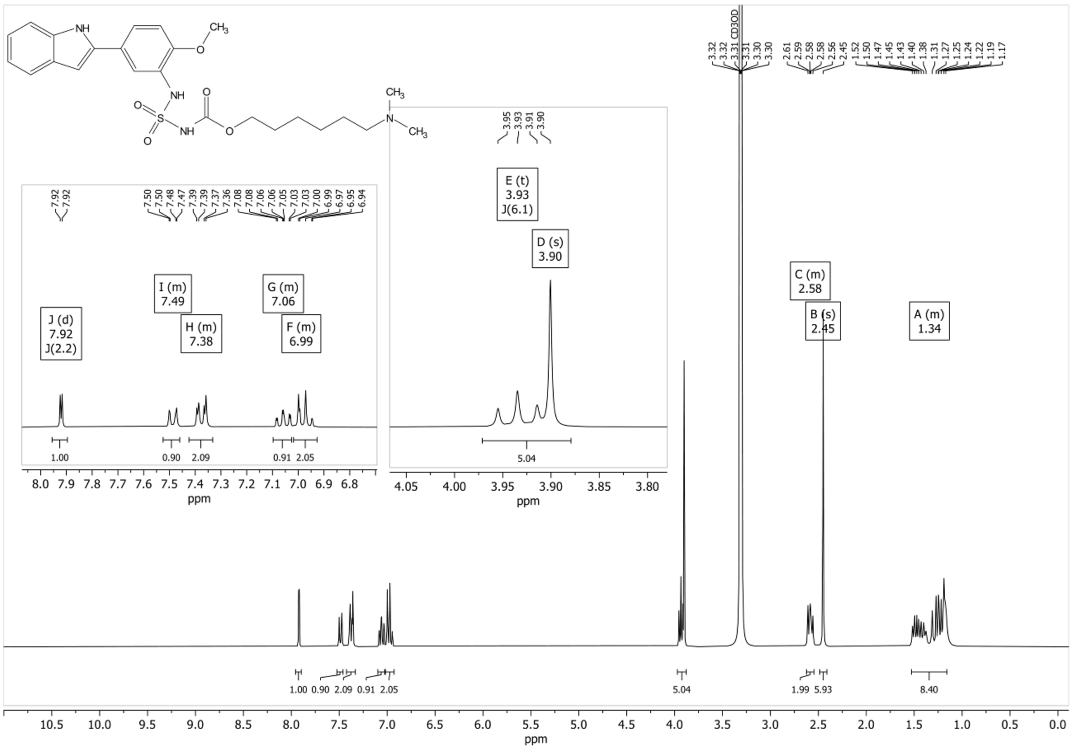

^{13}C NMR spectrum of compound **4a**

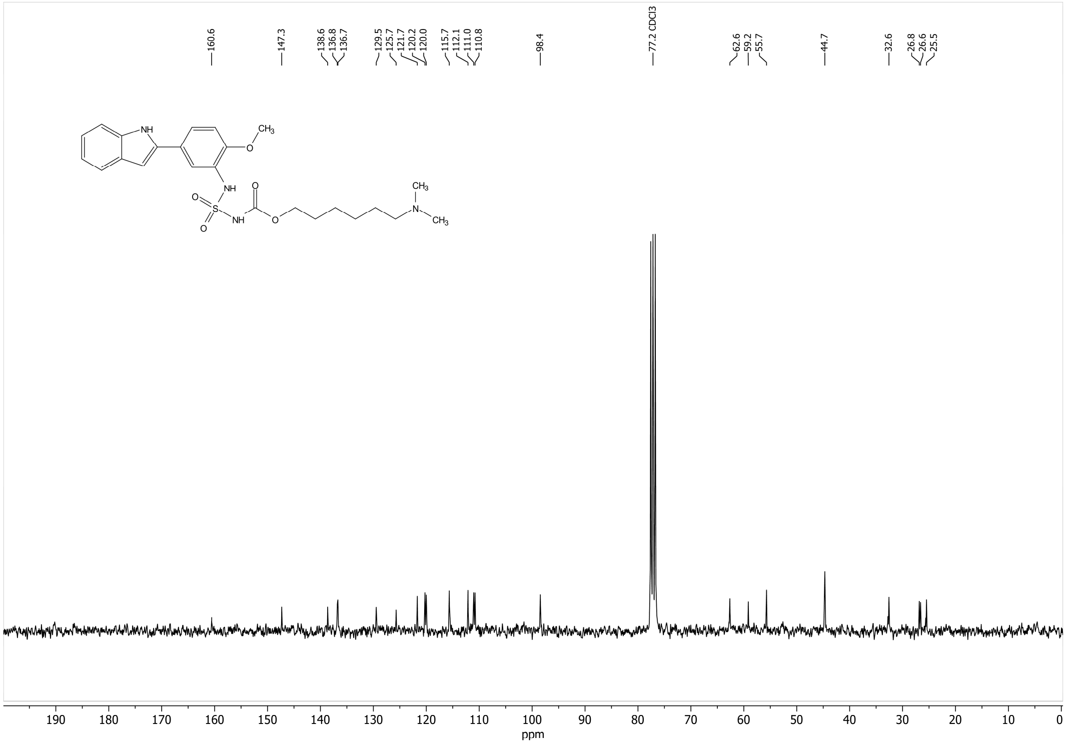

MS spectrum (ESI) of compound **4a**

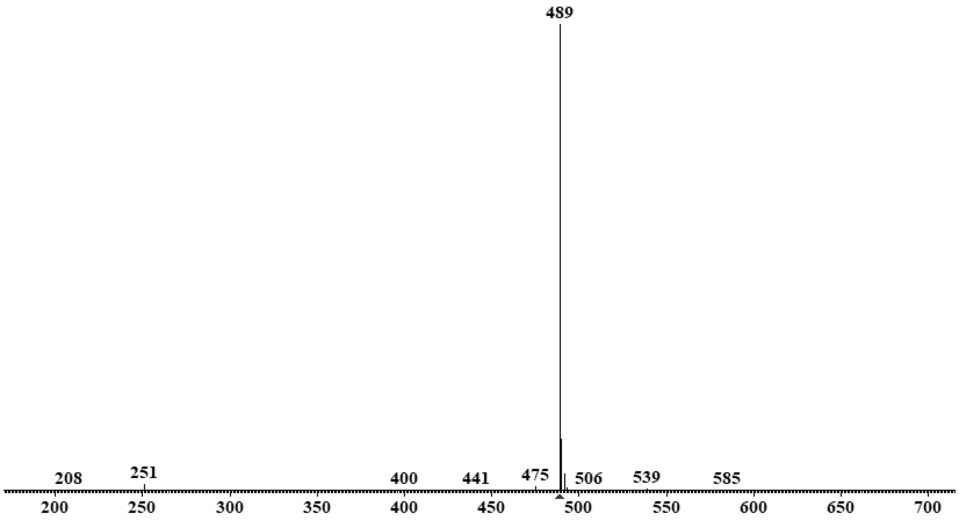

UV spectrum of compound **4a**

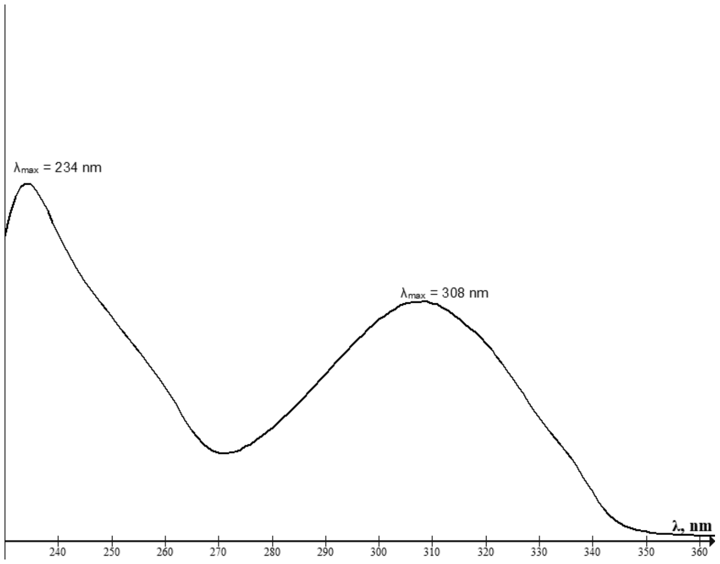

Analytical HPLC of compound **4a**

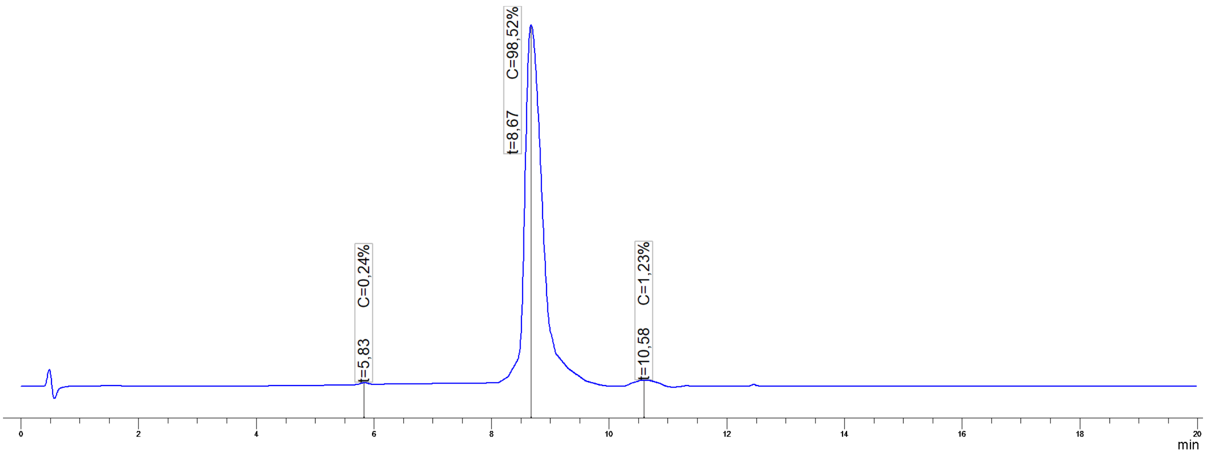

Gradient elution system composed of solvents A (H_2O with 0.1% formic acid and 10 mM ammonium formate) and B (ACN with 0.1% formic acid) at a flow rate of 0.6 mL/min was used. Mass spectrometric detection was performed using electrospray ionization (ESI).

1-Trimethylsilyl triethylene glycol (mono-TMS-TEG) (15)

^{1}H NMR spectrum of compound **15**

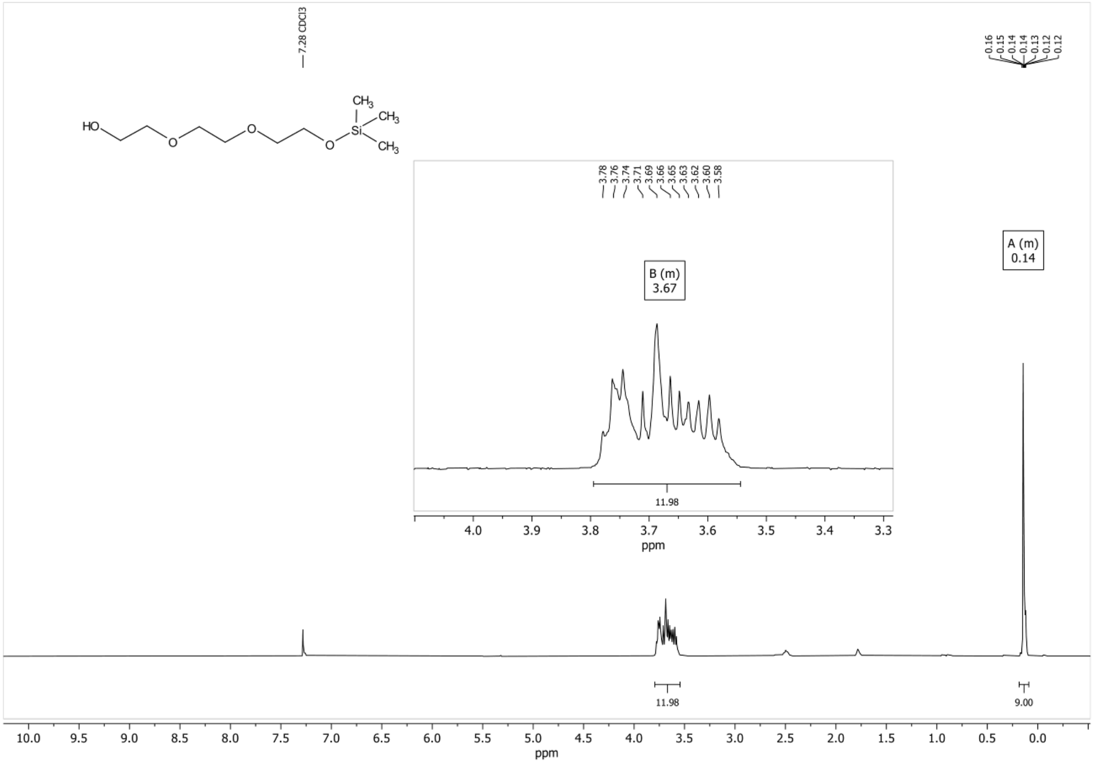

^{13}C NMR spectrum of compound **15**

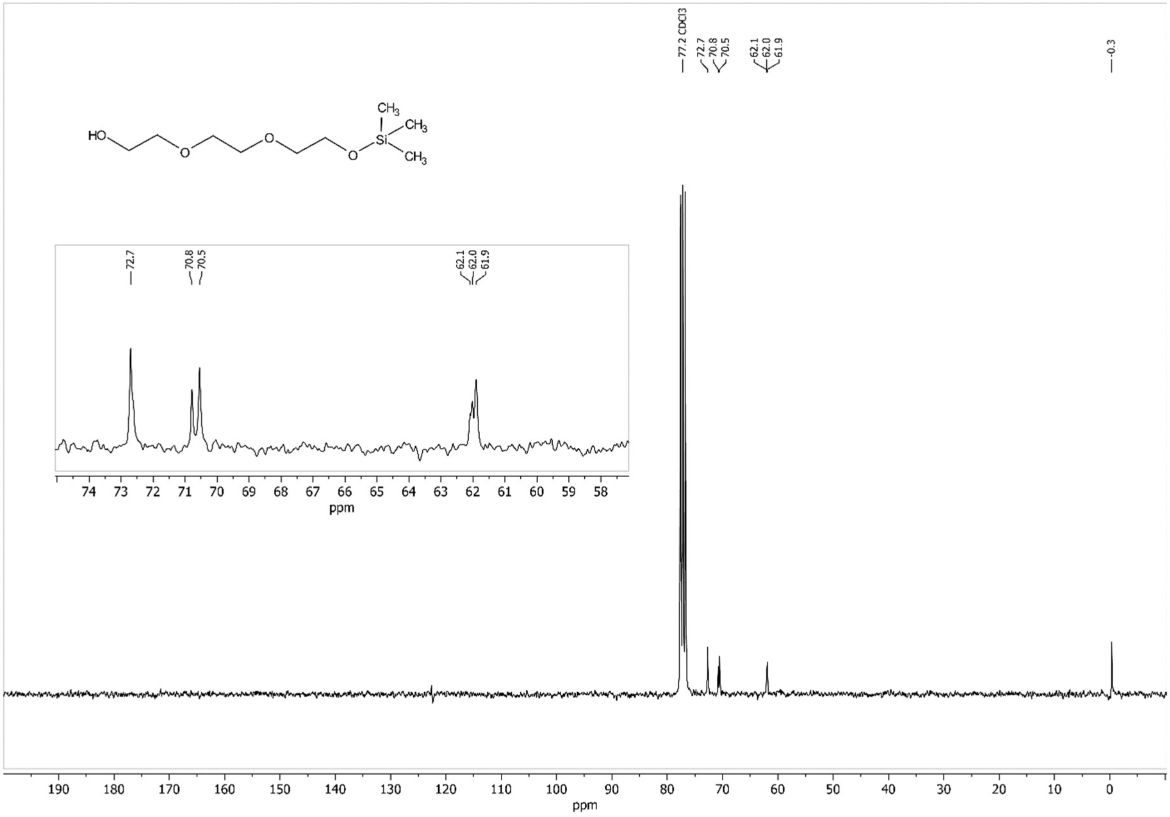

MS spectrum (EI) of compound **15**

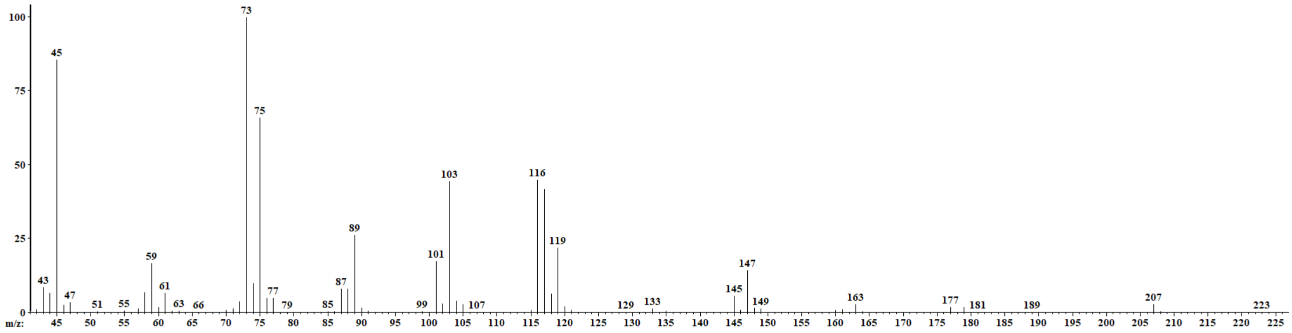

2-(2-(2-Hydroxyethoxy)ethoxy)ethyl (N-(5-(1H-indol-2-yl)-2-methoxyphenyl)-sulfamoyl)carbamate (4b)

^{1}H NMR spectrum of compound **4b**

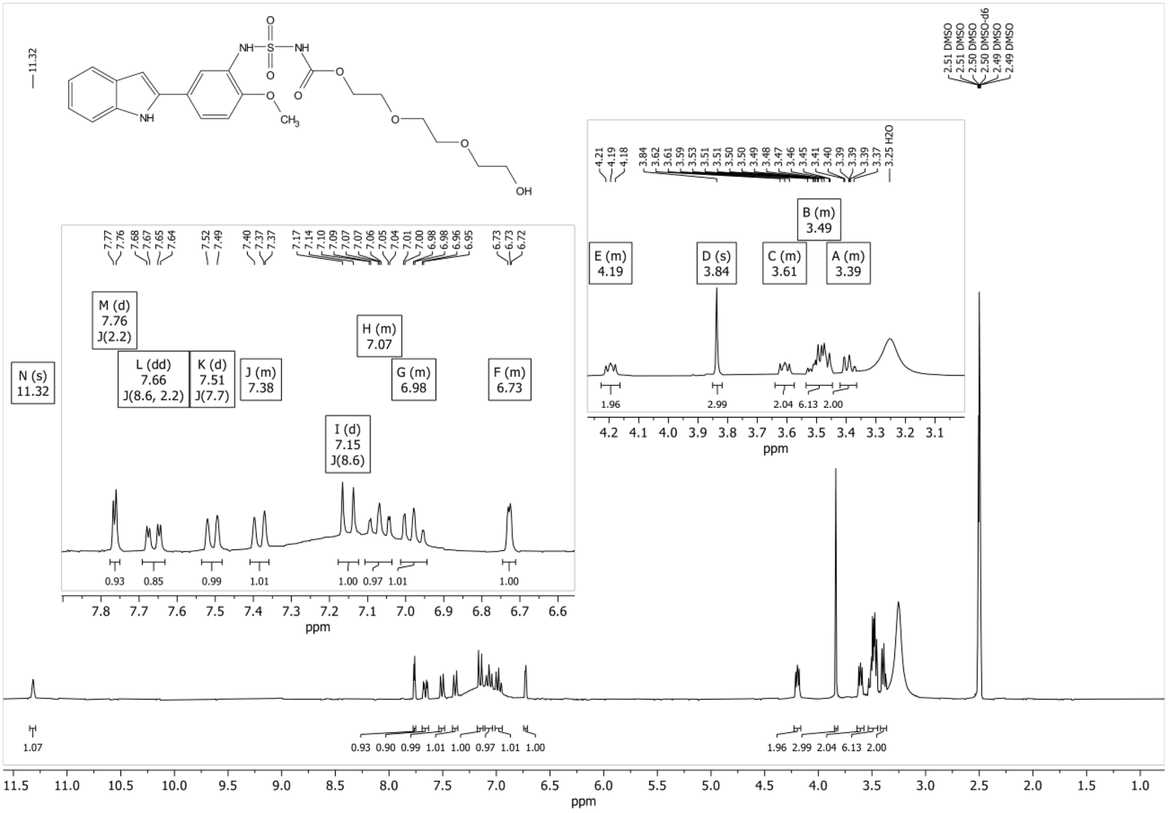

^{13}C NMR spectrum of compound **4b**

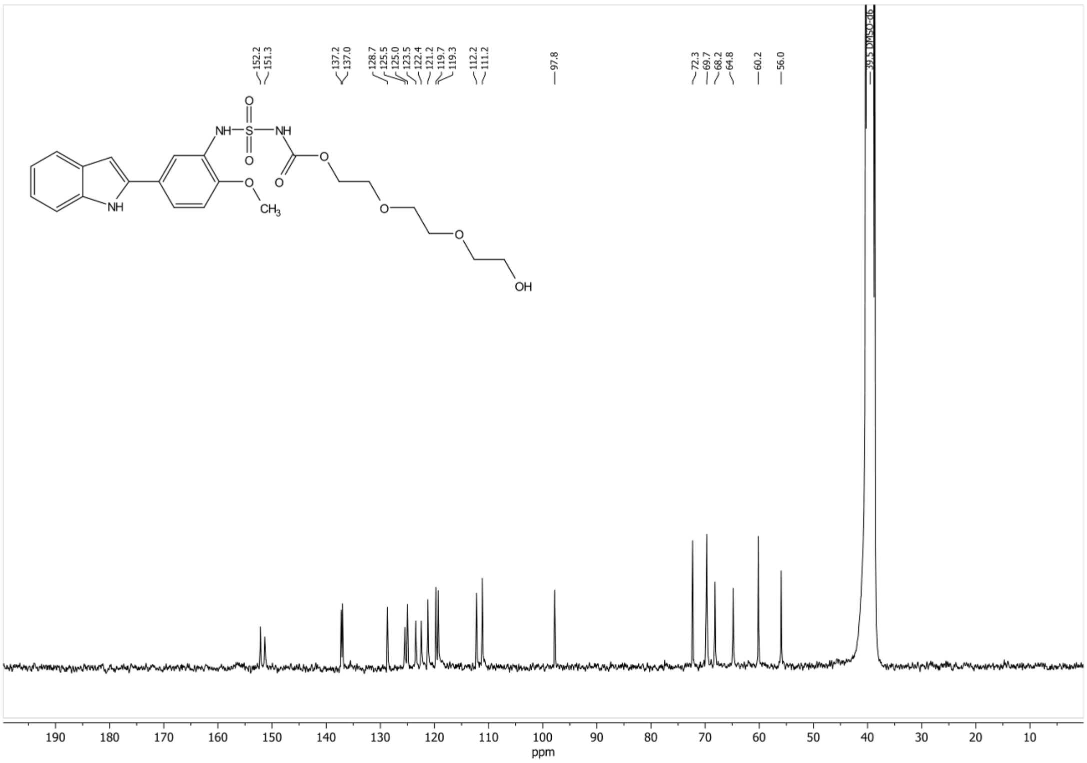

MS spectrum (ESI) of compound **4b**

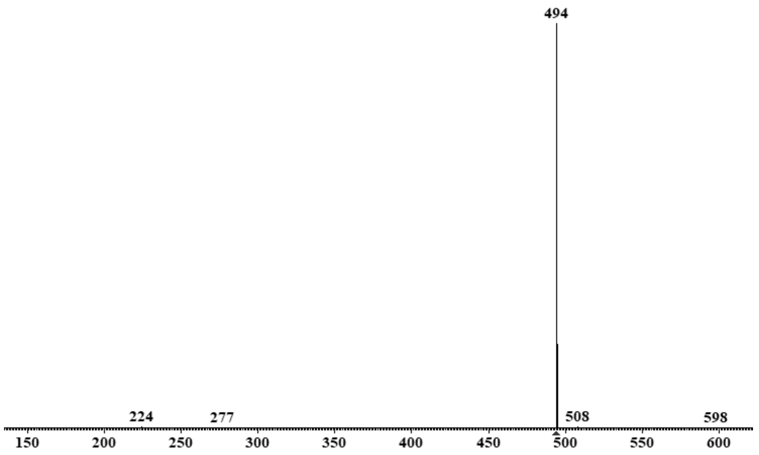

UV spectrum of compound **4b**

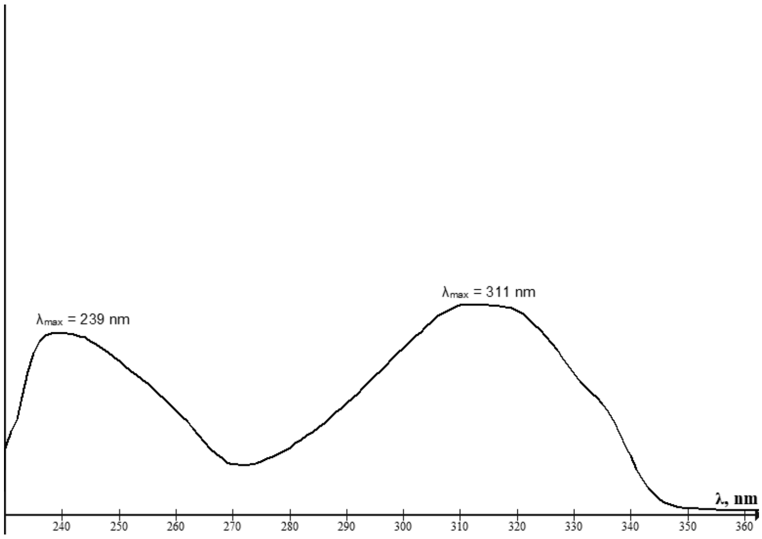

Analytical HPLC of compound **4b:**

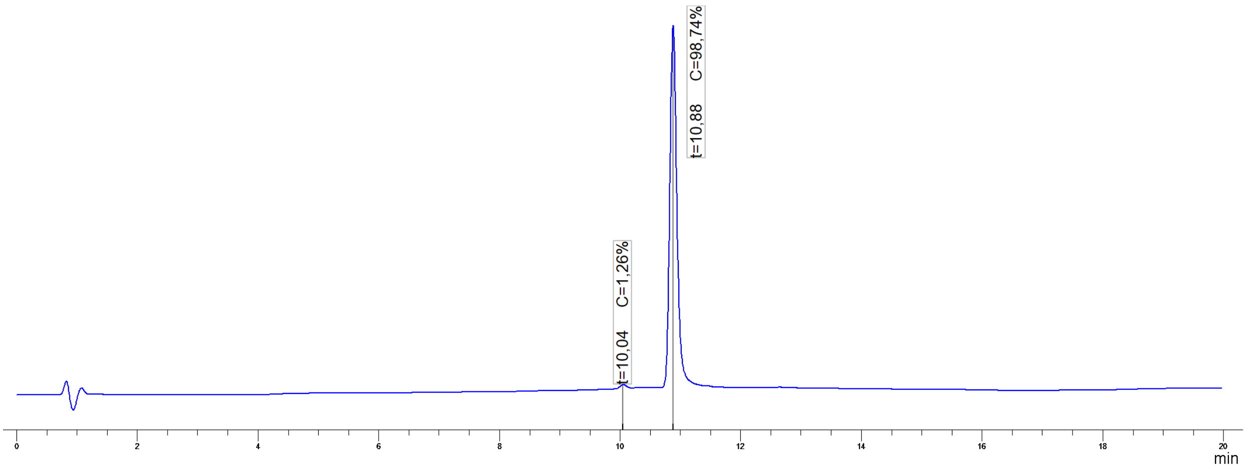

Gradient elution system composed of solvents A (H_2O with 0.1% formic acid and 10 mM ammonium formate) and B (ACN with 0.1% formic acid) at a flow rate of 0.6 mL/min was used. Mass spectrometric detection was performed using electrospray ionization (ESI).

Octyl methanesulfonate (18)

^{1}H NMR spectrum of compound **18**

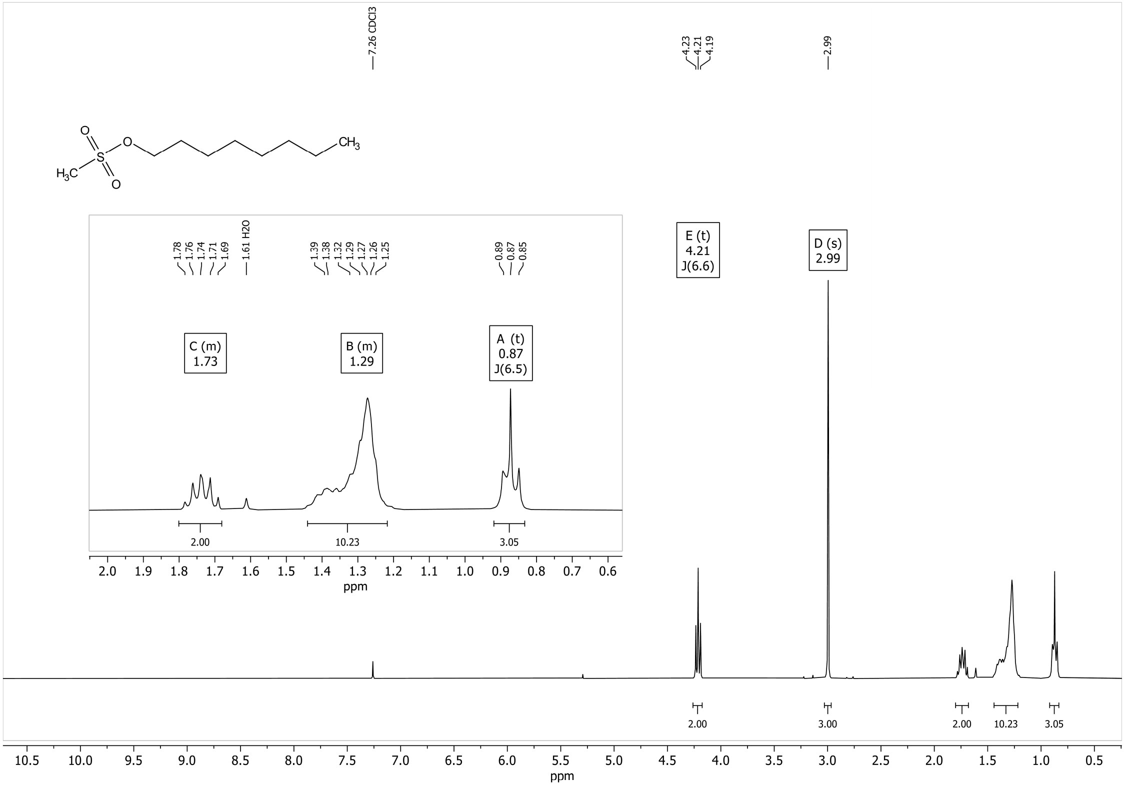

^{13}C NMR spectrum of compound **18**

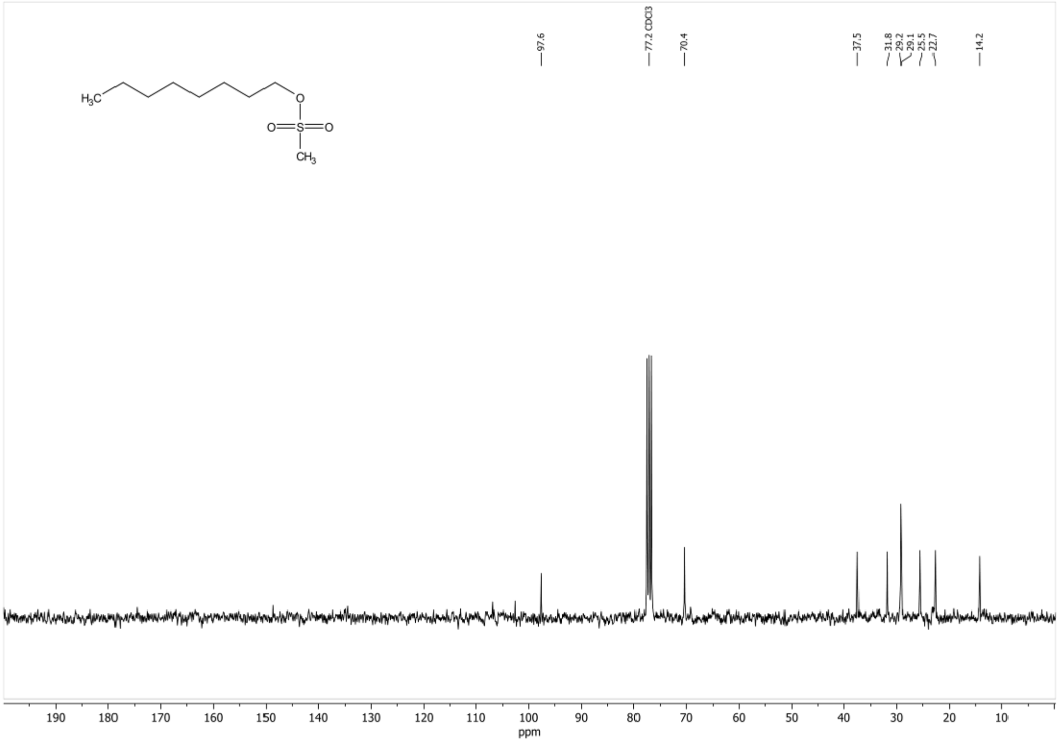

MS spectrum (EI) of compound **18**

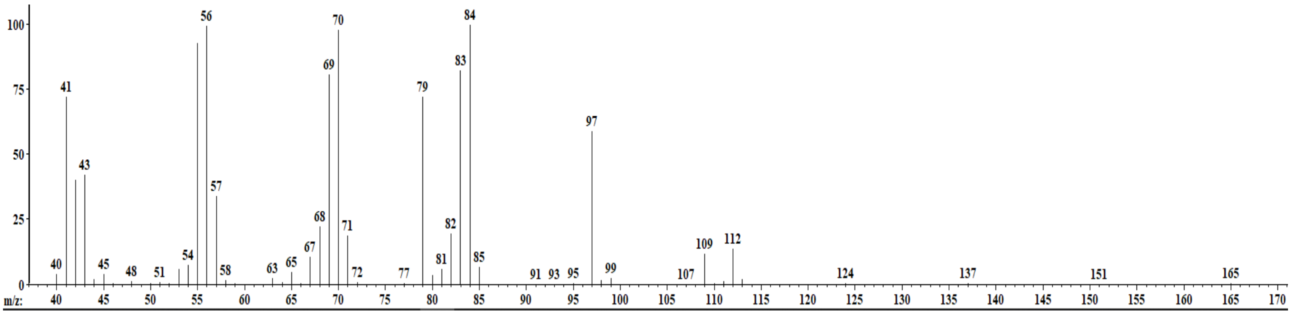

1-Iodooctane (19)

^{1}H NMR spectrum of compound **19**

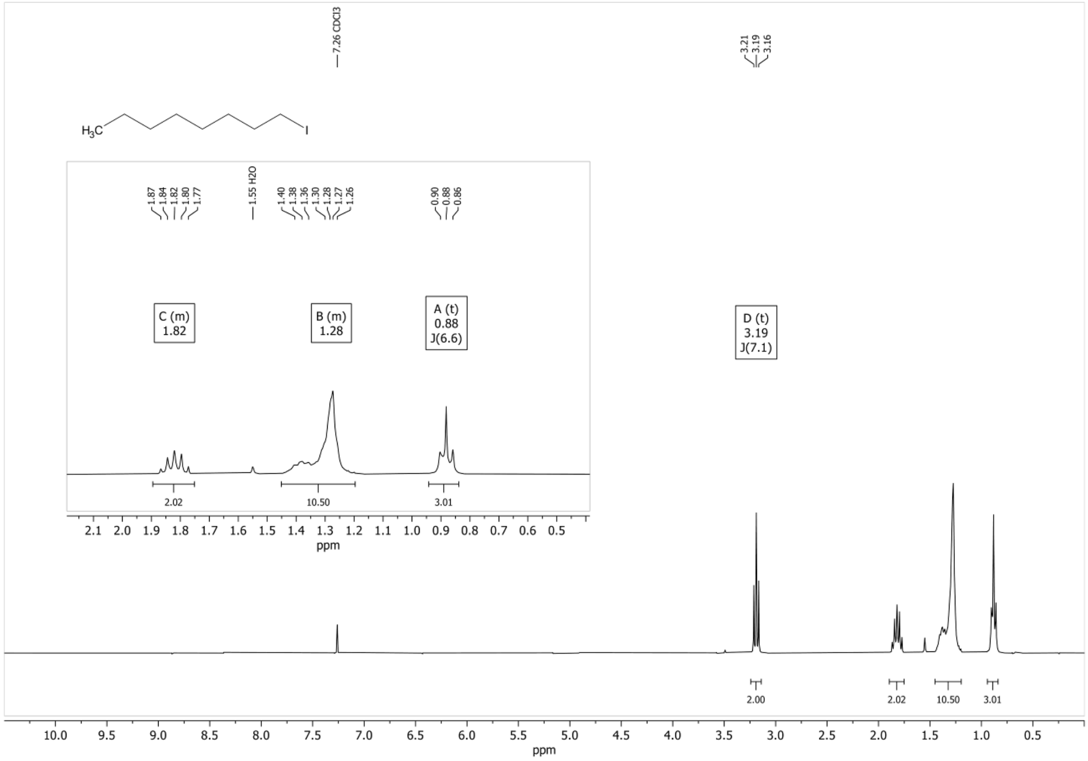

^{13}C NMR spectrum of compound **19**

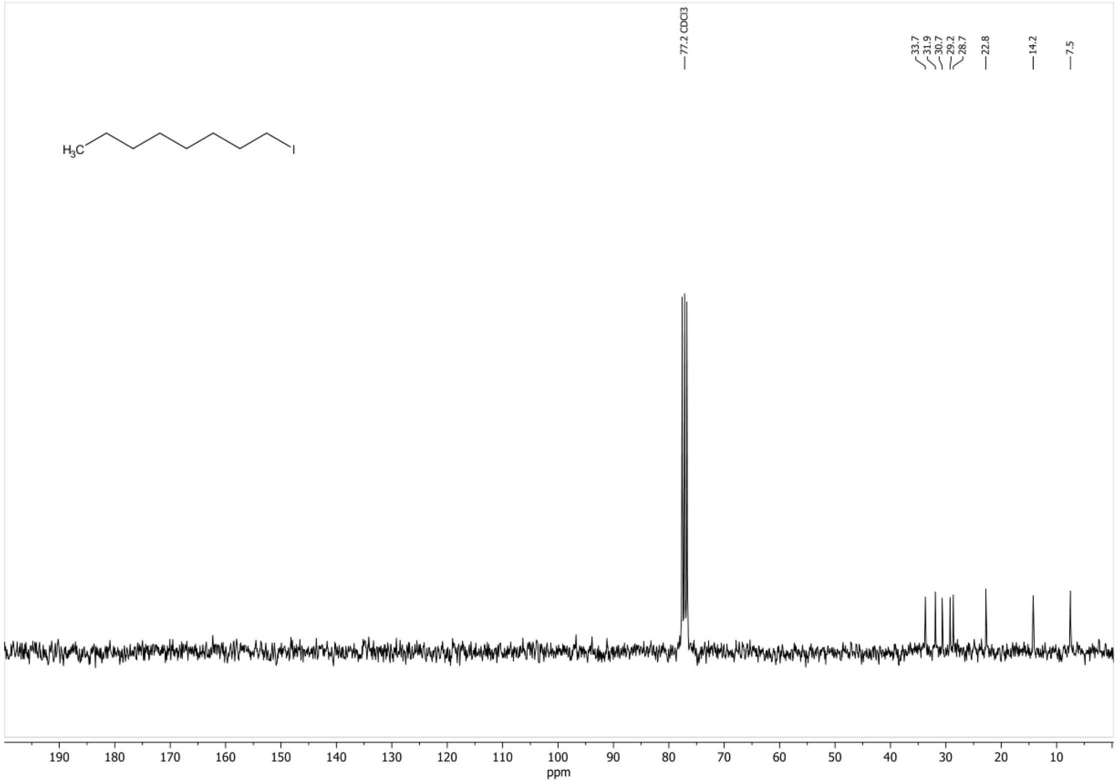

MS spectrum (EI) of compound **19**

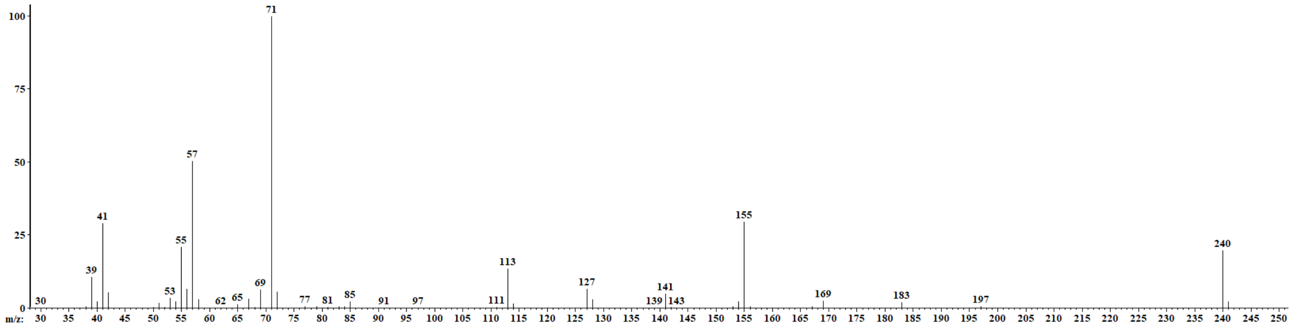

Sodium octane-1-sulfonate (20)

^{1}H NMR spectrum of compound **20**

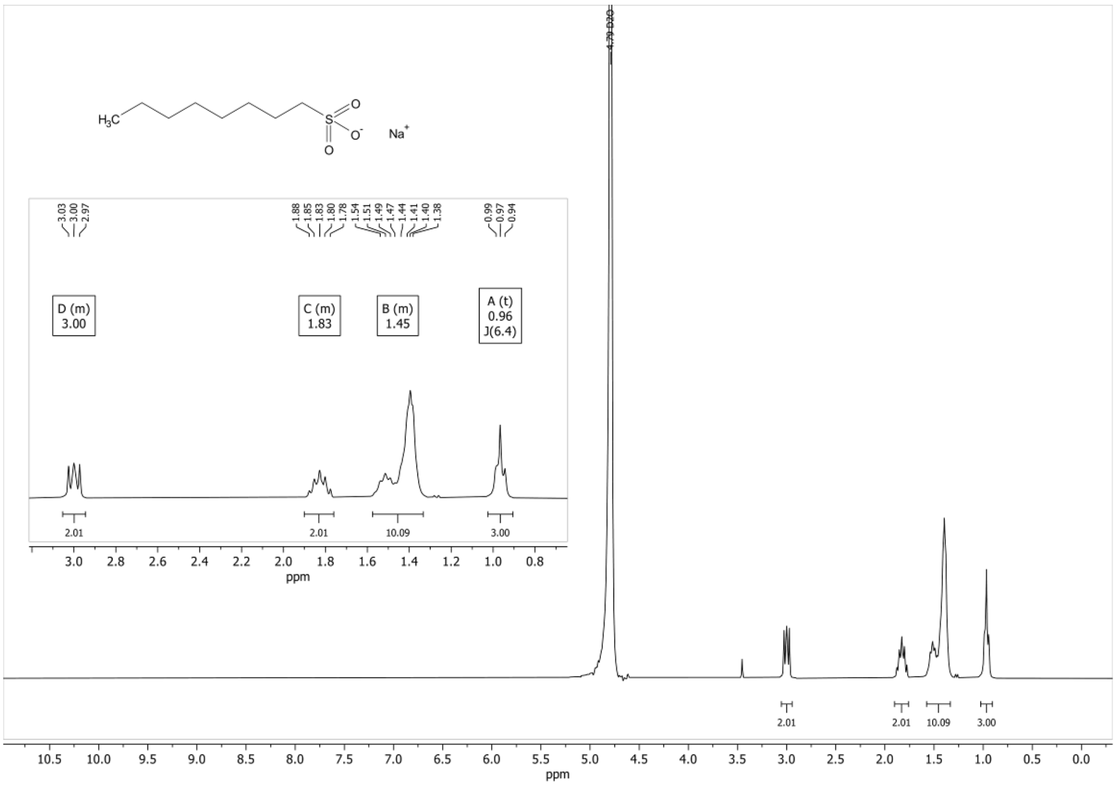

^{13}C NMR spectrum of compound **20**

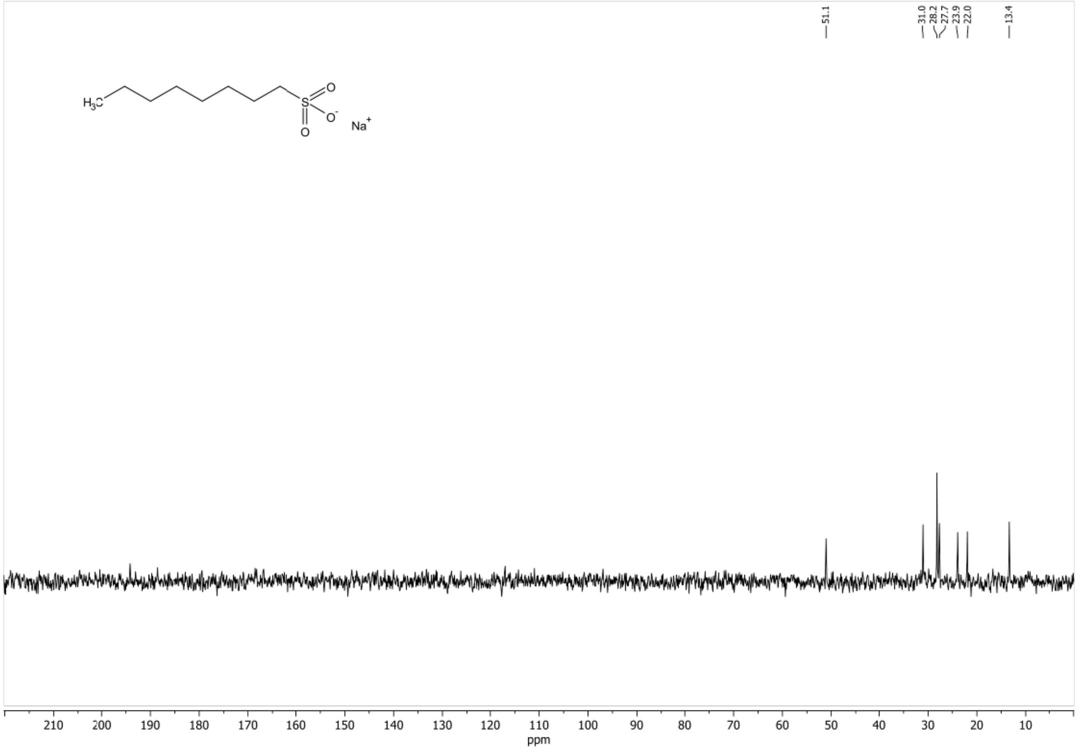

IR spectrum of compound **20**

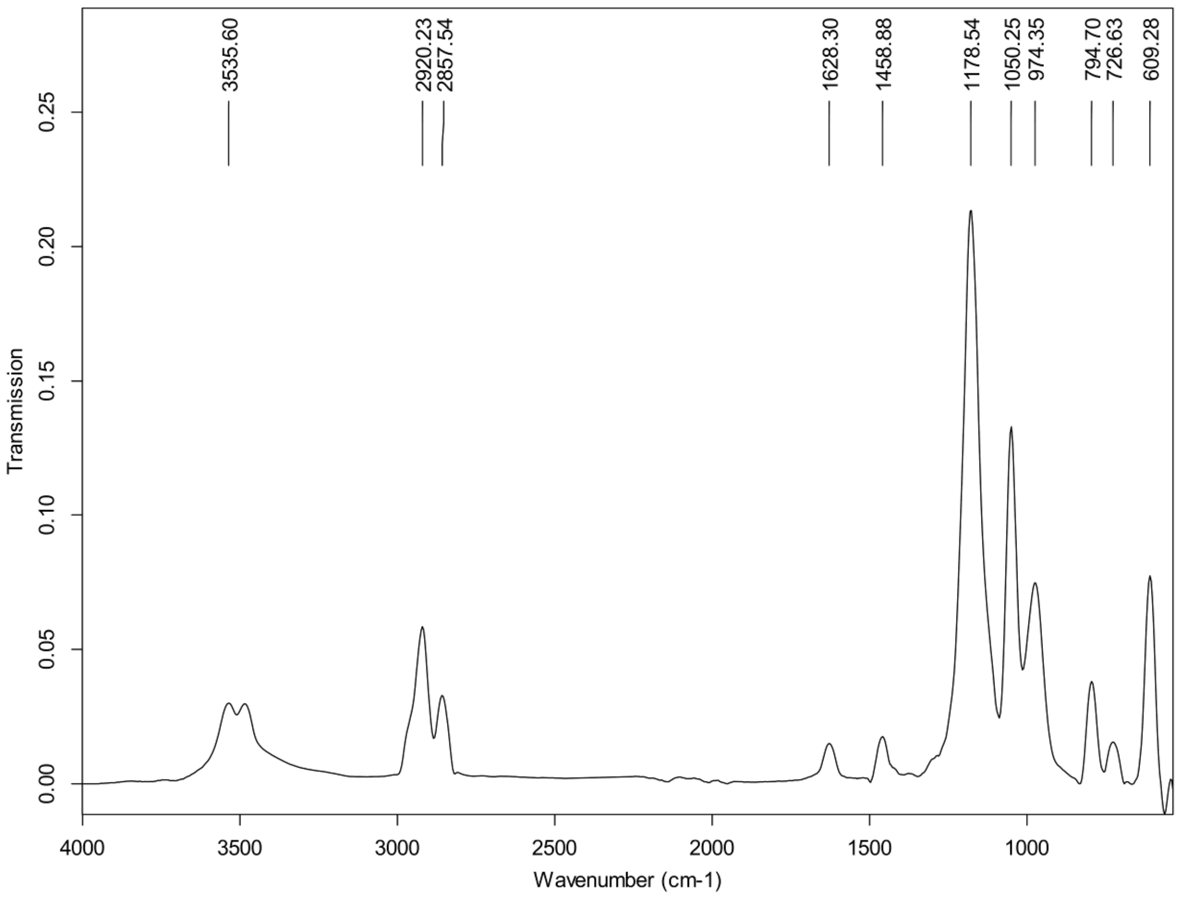

N-(5-(1H-indol-2-yl)-2-methoxyphenyl)-4-pentylbenzenesulfonamide (5a)

^{1}H NMR spectrum of compound **5a**

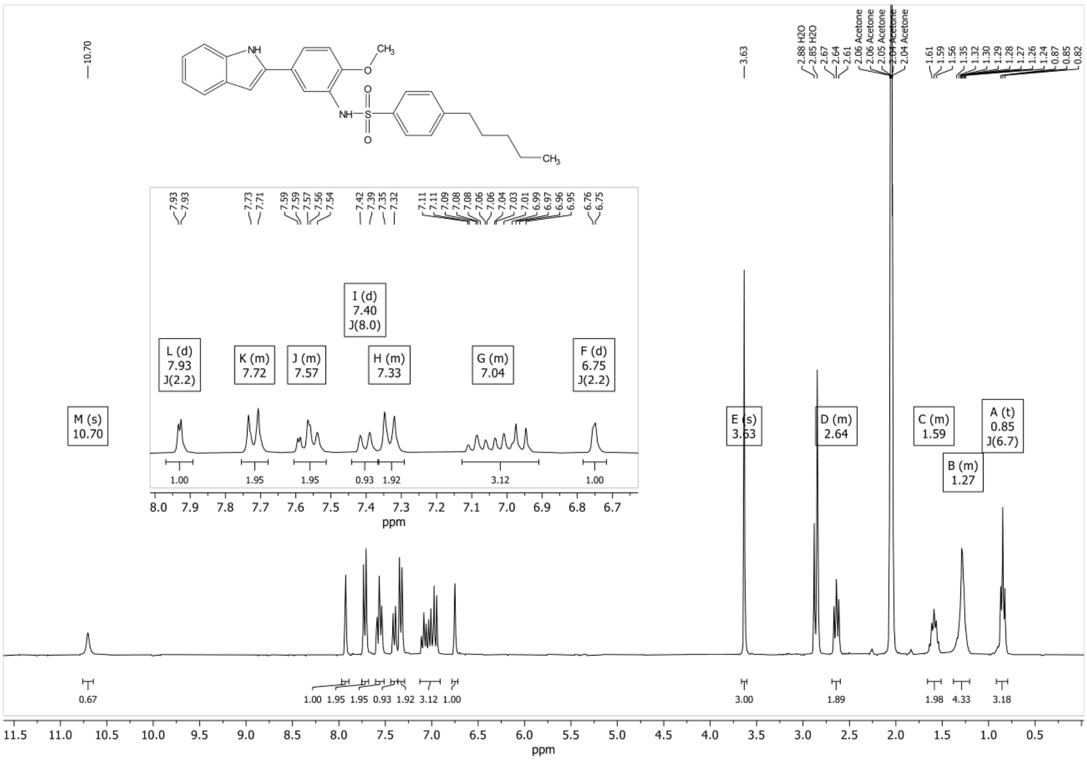

^{13}C NMR spectrum of compound **5a**

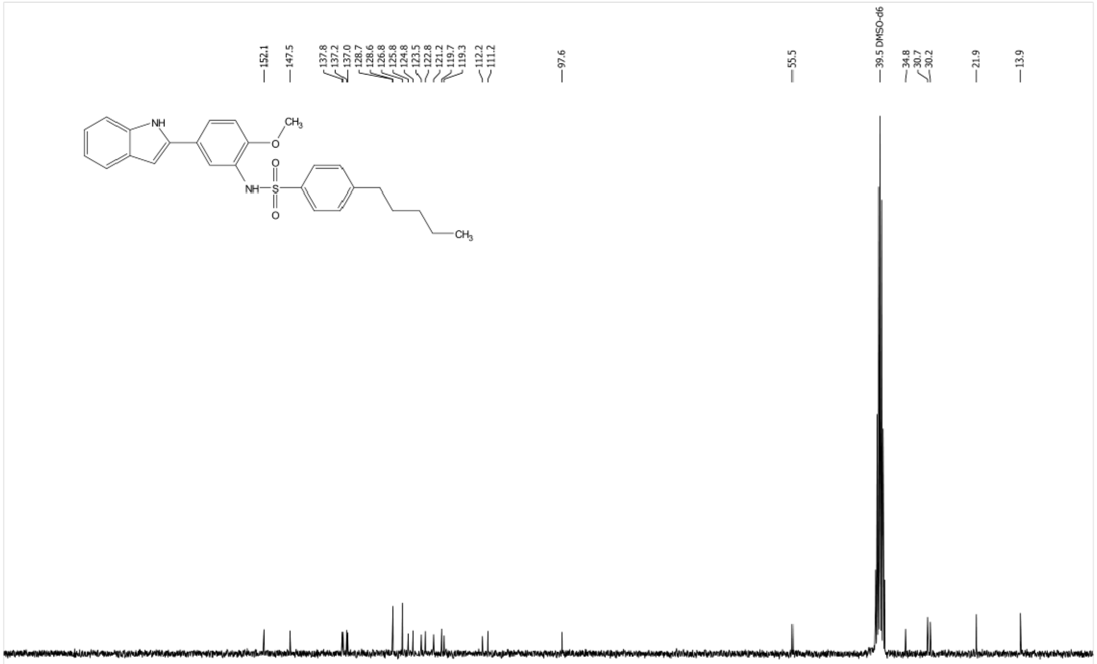

MS spectrum (ESI) of compound **5a**

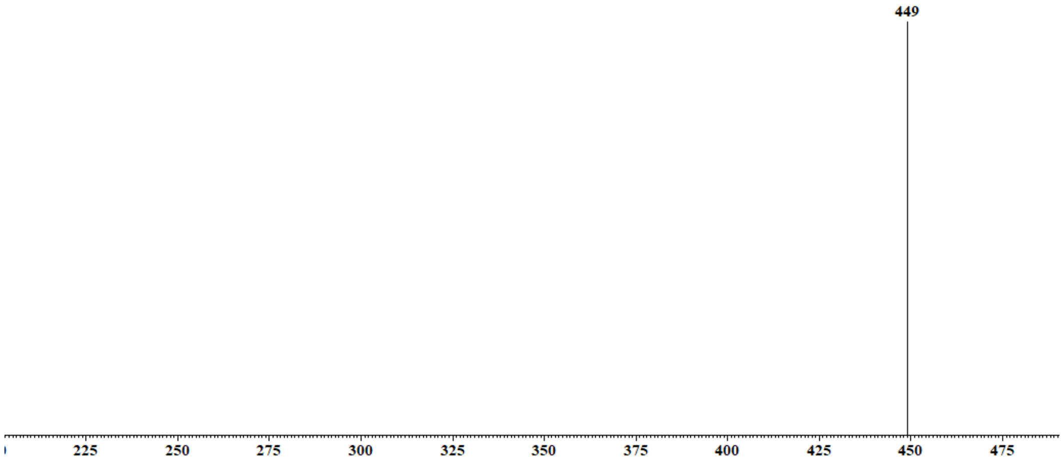

UV spectrum of compound **5a**

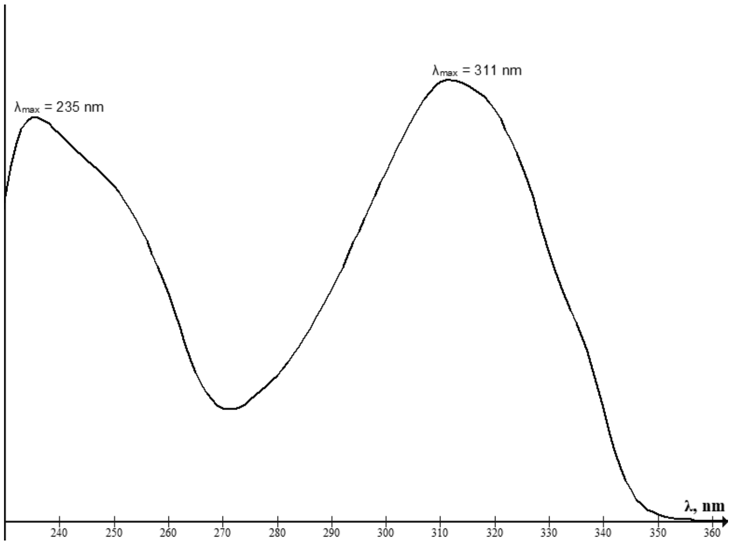

Analytical HPLC of compound **5a**

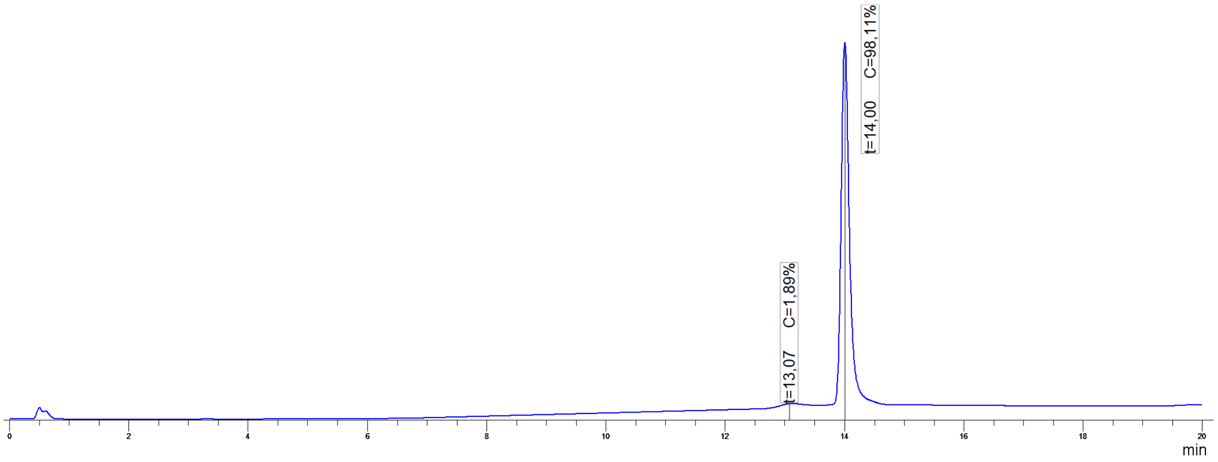

Gradient elution system composed of solvents A (H_2O with 0.1% formic acid and 10 mM ammonium formate) and B (ACN with 0.1% formic acid) at a flow rate of 0.6 mL/min was used. Mass spectrometric detection was performed using electrospray ionization (ESI).

N-(5-(1H-indol-2-yl)-2-methoxyphenyl)octane-1-sulfonamide (5b)

^{1}H NMR spectrum of compound **5b**

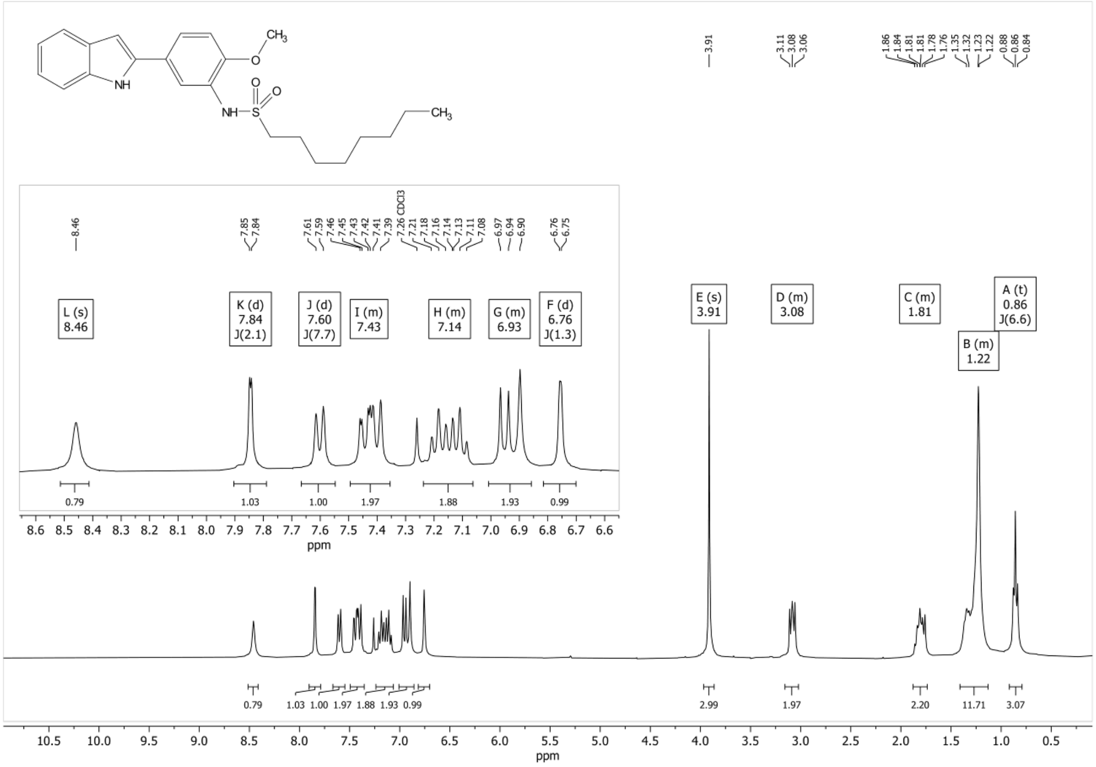

^{13}C NMR spectrum of compound **5b**

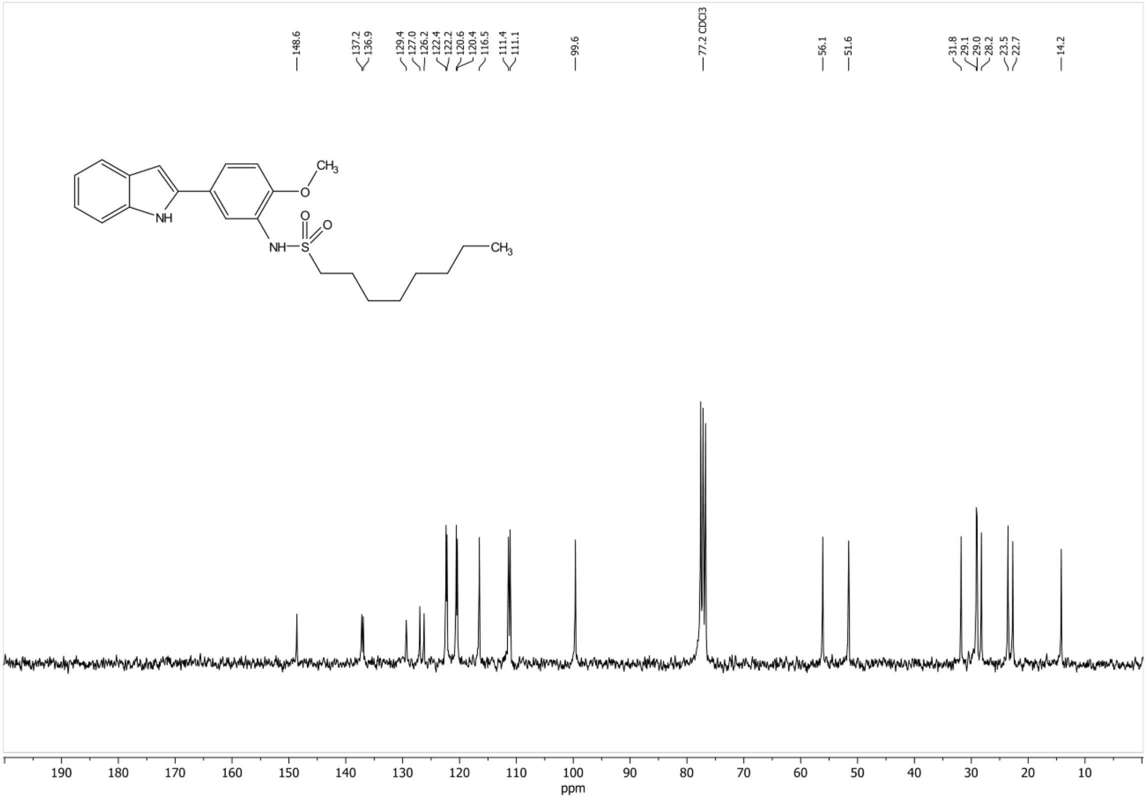

MS spectrum (ESI) of compound **5b**

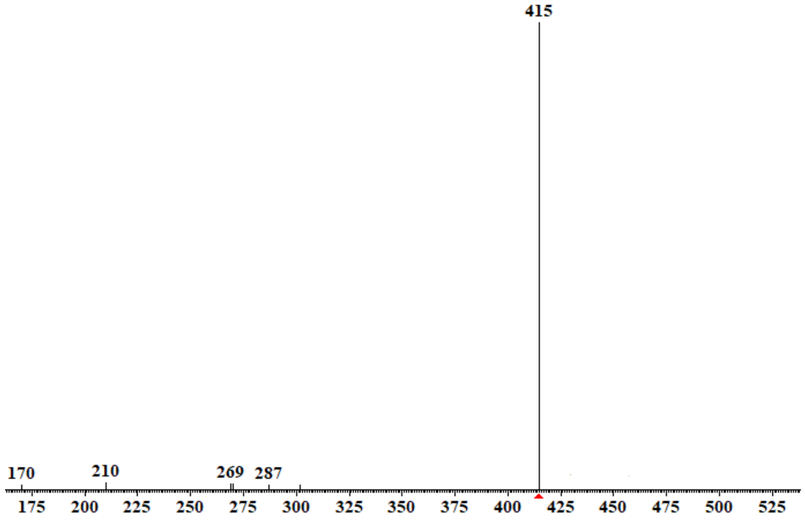

UV spectrum of compound **5b**

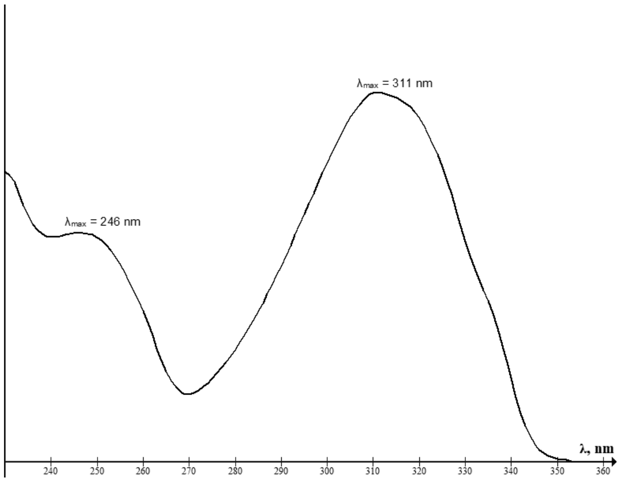

Analytical HPLC of compound **5b**

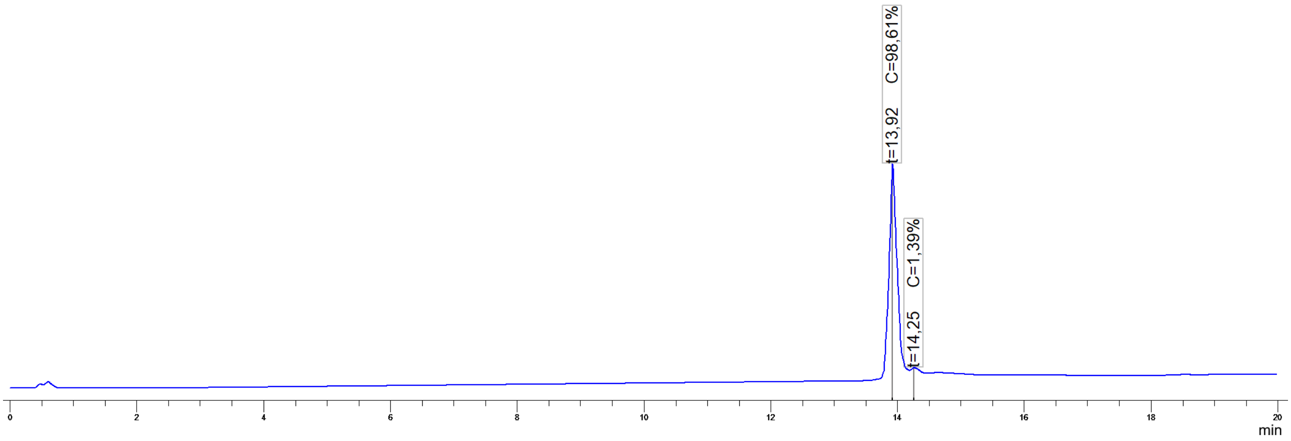

Gradient elution system composed of solvents A (H_2O with 0.1% formic acid and 10 mM ammonium formate) and B (ACN with 0.1% formic acid) at a flow rate of 0.6 mL/min was used. Mass spectrometric detection was performed using electrospray ionization (ESI).
